# Adjustment of a numerical model for pore pressure generation during an earthquake

**DOI:** 10.1371/journal.pone.0222834

**Published:** 2019-09-26

**Authors:** Jose Luis Garcia Diez, Jesus Gonzalez Galindo, Antonio Soriano Peña, for the Universidad Politécnica de Madrid

**Affiliations:** Department of Engineering and Soil Morphology, E.T.S. de Ingenieros de Caminos, C. y P., Universidad Politécnica de Madrid (UPM), Madrid, Spain; China University of Mining and Technology, CHINA

## Abstract

This article proposes methodology for evaluating the accuracy of the pore pressure generation model devised by Byrne, as implemented in a commercial software program using a Mohr-Coulomb-type failure criterion and a Finn constitutive model. The different empirical formulas of liquefaction developed by Seed and Idriss are reviewed, as well as various constitutive models specified in the literature, emphasizing the selection of the Finn model for the liquefaction study. In the analysis a comparison is carried out using the factors of safety against liquefaction (FSLs) devised by Seed and Idriss and the adapted formula by Boulanger and Idriss. The analysis assumes a hypothesis to verify whether a soil element is liquefied. The results are then compared with those of a numerical model that simulates a soil column, the base of which is subjected to the same seismic inputs of varying magnitudes, *M*_*w*_, and peak ground accelerations, *Pga*, to which the empirical model was subjected. Adjusted equations are provided on the based on that comparison to allow for the calibration of the Byrne equation using the *(N*_*1*_*)*_*60*_ value obtained via a standard penetration test (SPT), for the study of liquefaction problems in situations in which there are earthquakes of varying magnitudes.

## Introduction

In studies of soil dynamics, situations are presented that totally account for neither the adjusted response of a numerical calculation model with respect to the data input from field tests or site observations, or the relationship between the model and the field data. To this end, we aim to facilitate such studies by improving in the aforementioned data input scheme (in particular, with the *(N*_*1*_*)*_*60*_ value obtained from the SPT), correcting the values provided by a field study and soil characterization tests, to ensure a better fit to the formula stored in a computer program and provide a calculated solution with greater accuracy.

On this basis, new adjusted equations are provided to enable data input calibration in a behavior model within a numerical calculation model using the Byrne equation [1], by using the *(N*_*1*_*)*_*60*_ value obtained via the SPT, in situations in which earthquakes of varying magnitude occur.

Two summaries of previous studies, regarding the numerical models of soil behavior and the various empirical models devised by Seed and Idriss since 1971 are provided in detail below. Subsequently, a calculation adjustment is presented with the methodology proposed by the authors to provide the equations that will control the input data used to study of the behavior of granular soil, which is susceptible to liquefaction, against dynamic actions.

## Current state of knowledge

Liquefaction is defined as the loss of the shear resistance of soil subjected to monotonic or cyclical loads due to the tendency of less competent soils to reorganize their structure under shear stresses. The term was coined by Mogami and Kubo [2].

In many geotechnical projects, the model of Seed and Idriss is used to determine whether the soil can undergo liquefaction. Generally, if the results of the Seed and Idriss model indicate the possibility of liquefaction, some actions are carried out (soil improvements) to prevent it.

Geotechnical numerical models allow the total stresses to be calculated to determine if liquefaction occurs. The difficulty of using numerical models is in deciding how pore pressures are generated during the earthquake. To analyze, liquefaction using a numerical model, an adjustment to the equation of Byrne [1] is proposed to guarantee an improvement in modeling results.

If a simple case is analyzed (if a soil is liquefiable) by considering a specific earthquake, a certain discrepancy between the results obtained with the model of Seed and Idriss and those with a numerical model will arise. The purpose of this article is to propose a correction of the Byrne model that aligns the results of these two methods. This correction will allow more complex situations to be evaluated through numerical modeling.

### Empirical formula for the calculation of FSL

The state-of-practice simplified methods for the evaluation of liquefaction potential were developed using historical cases and field measurements at sites characterized with on-site tests (such as SPT). These simplified methods are generally expressed as a deterministic and semiempirical models.

In this approach, Seed and Idriss [3] concluded that the liquefaction of a soil would occur if FSL (assuming gently sloping ground with free-field conditions) is ≤1. FSL is defined as the relationship between the cyclic resistance ratio (CRR) and the cyclic stress ratio (CSR); therefore, if FSL>1, then the liquefaction of the soil would be unlikely.

Due to the use of field data collection during real earthquakes, researchers have progressively supplemented the initial Seed and Idriss formula [3] through, adaptations such as those shown in Table 1:

**Table 1:** Some adaptations of the empirical formula proposed to predict liquefaction and associated research.

Authors	Details of Advances
Seed and Idriss [3]	The beginning of the simplified procedure to evaluate the potential for soil liquefaction based on the FSL, relating the CRR and CSR.
Shibata [4]	New relationships between the *N* value and the liquefaction potential in sandy soil deposits.
Tokimatsu and Yoshimi [5]	Correlations between the *N* value and the content of fine sandy liquefiable soils.
Seed et al. [6]	Analysis of 126 data points from the case history employed to obtain correlations between the CSR and the *(N*_*1*_*)*_*60*_ value for earthquakes of *M*_*w*_*<7*.*5*, considering different fine soil contents.
Seed et al. [7]	Adaptations of case studies with the new adjustments of the formula and the *r*_*d*_ factor.
Golesorkhi [8]	Development of the proposed formula such as the coefficients in CRR and CSR.
Idriss [9]	Extension of the work of Golesorkhi [8] indicating that the parameter *r*_*d*_ could be obtained as a function of the soil depth and earthquake magnitude.
Cetin et al. [10]	Probabilistic and statistical analyses that refer to real case studies to adjust the *r*_*d*_ factor.
Youd et al. [11]	Determination of the differences between the liquefaction triggering correlations published by Seed et al. [6–7].
Cetin et al. [12]	Diverse adjustments of the CRR formula for an earthquake of *M*_*w*_ *= 7*.*5* and *σ'*_*v*_ *= 1 atm* vs. *(N*_*1*_*)*_*60cs*_, with lower values than Seed et al. [6].
Idriss and Boulanger [13]	Adjustments of the CRR formula for an earthquake of *M*_*w*_ *= 7*.*5* and *σ'*_*v*_ *= 1 atm* vs. *(N*_*1*_*)*_*60cs*._
Idriss and Boulanger [14]	Adjustments of the CRR and CSR formulas for an earthquake of *M*_*w*_ *= 7*.*5* and *σ'*_*v*_ *= 1 atm* vs. *(N*_*1*_*)*_*60cs*._
Boulanger et al. [15]	Updates to case history databases and adjustment of the formula using SPT values.
Idriss and Boulanger [16]	Updates to case history databases. More detailed illustrations of the database distributions related to liquefaction are provided. Presentation of a probabilistic version of the liquefaction trigger according to Idriss and Boulanger [13–14]. In addition, new findings in the analysis of liquefaction.
Boulanger and Idriss [17]	The procedures based on the SPTs of Youd et al. [11] and Idriss and Boulanger [16] are compared to the case history data in [16], adjusted to the effective vertical stress equivalent to *σ*_*v*_ *= 1 atm* and an earthquake of moment magnitude of *M*_*w*_ *= 7*.*5*. New calculation correlations are obtained.
Cetin et al. [18]	A concise summary of the improved database and the updated liquefaction activation relationships, readjusting the values of CRR, is presented.
Cetin et al. [19]	Adaptation and updating of the work developed by Cetin et al. [10–12]. Presentation of new curves based on SPT tests.
Yang et al. [20]	Proposal for a new formula to evaluate liquefaction in sandy layers in depths of 10 to 20 m.
Rostami et al. [21]	Incorporation of a seismic energy attenuation model to re-evaluate a liquefaction history database based on the SPT tests.

Analyzing the previously published data and observing the recent research detailed in the previous table, the advances developed by Boulanger and Idriss [17] were selected to be used in the empirical formulation for the calculation of liquefaction potential. The authors find that the details of these updates to the CSR formulas are the most appropriate.

### Constitutive models of soil subjected to dynamic loads

The study of soil dynamics has been addressed in recent years by a large number of researchers. These works provide constitutive models that integrate both the characteristics of the soils (damping and resistance to cyclic loads) and the properties of the boundary conditions (site).

Over time, numerical models, based on the global constitutive models, have been developed in the study of soil dynamics problems and are incorporated in specialized calculation programs. Some of these models are detailed in Table 2:

**Table 2:** Compilation of dynamic models applied to the objective of this study.

Authors	Model Name
Wang et al. [22]	Wang 2D
Martin et al. [23] and Byrne [1]	Finn
Jefferies [24]	Nor-Sand
Byrne et al. [25]	UBC Sand
Rauch and Martin [26]	EPOLLS
Galindo [27], based on the tests by Patiño [28]	R. Galindo
Adrianopoulos et al. [29]	NTUA Sand

Over the past few years, the Research Group of the Department of Engineering and Ground Morphology of the Advanced School of Civil Engineering (ETSICCP, as per its Spanish acronym) at the Technical University of Madrid (UPM, as per its Spanish acronym), has made advances in numerical modeling, especially with the Finn model (with Byrne equation), which is the model most adjusted to suit the work of the Research Group.

The development of the study and calibration of the Finn model is reflected in the analysis of Galindo [27] on the laboratory cyclic shear tests carried out by Patiño [28], conducted under different combinations of stresses representing dynamic loads at different frequencies, and then applied to a real case study by Soriano [30]: the sinking of some concrete caissons in the mouth of the Port of Barcelona due to the liquefaction of the foundation sands.

The Finn model is defined by an equation (of volumetric deformation) that depends on the *(N*_*1*_*)*_*60*_ value from a SPT test, with allows for the calculation of pore pressure generation, unlike the empirical method that utilizes the formulation of Boulanger and Idriss to determine the occurrence of liquefaction. Therefore, the only parameter that could be varied was the N of the SPT.

Therefore, the Finn model, implemented in the explicit finite difference program FLAC3D, is chosen to carry out the study presented in this article.

#### Finn Model

Martin et al. [23] described the pore pressure generation mechanism of this model, highlighting that the relationship between the irrecoverable volume deformations and the range of the cyclic shear strains depends on the confining stress.

Based on the work of Martin et al. [23], Byrne [1] established a more simple formula for the Finn model that depends solely on one value of the soil, the *(N*_*1*_*)*_*60*_ value, which takes into account the usual parameters (angle of internal friction, deformation modulus, etc.). These two formulas are intrinsically related; in fact, if the exponential term of Byrne’s formula was written with reference to the first two terms of the Taylor series expansion, Byrne’s model would be the same as Martin’s formula.

This model, implemented in FLAC3D, the explicit finite difference program, is chosen to carry out the study presented in this article.

## Proposed methodology

An improvement in the numerical modeling used to perform dynamic calculations in liquefiable soils is proposed by adjusting the Byrne equation. In particular, the input of the *(N*_*1*_*)*_*60*_ value of the SPT test is modified here because it is the fundamental parameter of the Byrne model.

To this end, the empirical method of global reference and the Finn model with the formulation of Byrne are used, with the data provided by nine seismic inputs with different *M*_*w*_ and *Pga* values, to establish the generation of pore pressures within the FLAC3D environment and the FSL of Seed and Idriss (adapted by Boulanger and Idriss).

The following development plan, based on equalizing the FSLs to obtain the adjusted Byrne formula [1], is presented:

- Selection of a group of representative seismic inputs to be applied to the base of the model; each group is characterized by its moment magnitude (*M*_*w*_) and by its peak ground acceleration (*Pga*).
- Determination of the *(N*_*1*_*)*_*60cs*_ value for each soil element with FSL = 1, according to the formulation of the liquefaction model by Seed and Idriss [3], adapted by Boulanger and Idriss [17]. These factors are all dependent on the features of the input acceleration and the depth of the soil element considered.
- Formulation of the numerical model and assignment of the soil properties considered using the *(N*_*1*_*)*_*60cs*_ value obtained in the second section.
- Solution of the numerical model for each seismic input and the subsequent obtainment of the defined FSL as the coefficient between the effective mean stress before the earthquake $\sigma_{m}^{\prime }(t_{0})$ and the excess pore pressure immediately after: Δ*pp* = *pp*(*t*_*f*_)-*pp*(*t*_0_).
- If the achieved results are not satisfactory, i.e., if the obtained FSL, according to the previously described definition, is not equal to 1, an iterative calculation is carried out to determine to the *(N*_*1*_*)*_*60cs*_ value, which guarantees FSL = 1.

### Selection of input accelerograms

Nine seismic inputs were selected with a moment magnitude of *M*_*w*_ *≥ 6*.*5*, given that lower magnitudes and the associated peak ground accelerations commonly require *(N*_*1*_*)*_*60cs*_ values that are extremely low and cause liquefaction in soils. Three moment magnitudes are considered, 6.5, 7.5, and 8.5, each with low, medium and high peak ground accelerations. In Table 3, the records used and their main features are shown.

**Table 3:** Seismic events used and their basic associated properties.

Earthquake Considered		Date of Occurrence	Station	M_w_	Pga (m/s^2^)	Database [31]
1	Central Italy (CI)	30/10/2016	Matelica	6.60	1.22	European Strong-Motion Database
2	San Fernando (SF)	02/09/1971	LA-Hollywood Stor FF	6.61	2.00	Peer NGA Strong-Motion Database
3	Imperial Valley (IV)	15/10/1979	Bonds Corner	6.53	5.90	Peer NGA Strong-Motion Database
4	Tabas-Iran (TI)	16/09/1978	Boshrooyeh	7.35	0.85	Peer NGA Strong-Motion Database
5	Kern County (KC)	21/07/1952	Taft Lincoln School	7.36	1.60	Peer NGA Strong-Motion Database
6	Puerto Quellon (PQ)	25/12/2016	Hotel Espejo de Luna	7.50	3.50	Strong-Motion Virtual Data Center (VDC)
7	Maule (M)	27/02/2010	Valdivia	8.80	1.40	Strong-Motion Engineering Data Center (EDC)
8	Coquimbo (CO)	16/09/2015	Obs. Tololo	8.30	3.40	Strong-Motion Engineering Data Center (EDC)
9	Coquimbo (CP)	16/09/2015	El Pedregal	8.30	6.70	Strong-Motion Engineering Data Center (EDC)

**Fig 1:**
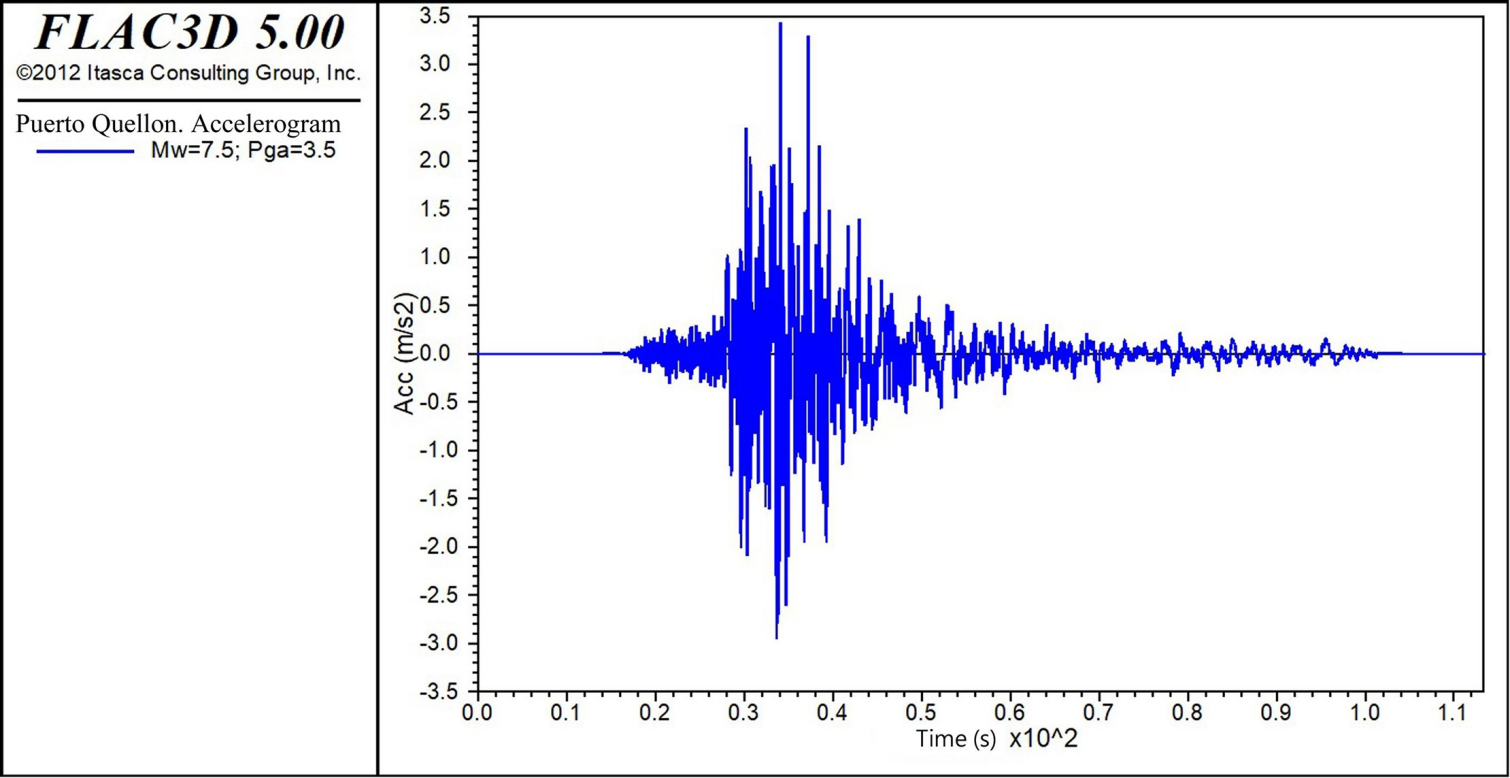
Puerto Quellon (PQ) input accelerogram.

All the records were originally filtered for low frequencies, generally lower than 0.1 Hz. In addition, their baselines have been corrected. Fig 1 shows one of the nine FLAC3D input accelerograms used, that of Puerto Quellon (PQ).

### Application of the seed and Idriss model (1971), adapted by Boulanger and Idriss (2014), and the attainment of *(N*_*1*_*)*_*60cs*_

In liquefaction risk assessments, it is customary to use the Seed and Idriss model (1971) or the subsequent updates, as these provide an FSL that describes the possibility of a particular granular soil (characterized by *(N*_*1*_*)*_*60cs*_) liquefying during a seismic stimulation with a certain moment magnitude, *M*_*w*_, and peak ground acceleration, *Pga*, when FSL<1. The denomination of the *(N*_*1*_*)*_*60cs*_ value stems from the denomination of Seed and Idriss of the corrected *(N*_*1*_*)*_*60*_ value of the SPT and with the added “*cs*” detail, referring to the adjustment for “*clean sand*”. Therefore, the same name is used in this article.

The Seed and Idriss model is based on the concept of resistance to cyclic loading, using the CRR, the maximum tangential stress of a soil sample before reaching the yield strength in a cyclic simple shear test is, confined under a determined effective vertical stress and a cyclic strain is applied with a given frequency.

It is generally accepted that earthquakes are multifrequency signals. Nevertheless, Seed and Idriss find a certain relationship between the application frequency of cyclic loading and the moment magnitude. Therefore, the CRR parameter can be defined as a function of the features of the sample, its confinement and the predominant frequencies.

With cyclic resistance defined, cyclic loading must be defined such that the relationship between them provides the FSL. In this sense, Seed and Idriss define cyclic loading, or CSR, as the maximum shear stress that a soil element undergoes during an earthquake due to the maximum acceleration. Given that the maximum acceleration is not directly representative of the average effect of the earthquake, Seed and Idriss [3], with the formula adapted by Boulanger and Idriss [17], adjusted this value with a coefficient of 0.65. Furthermore, this model is based on one soil column that behaves as a rigid body; therefore, a parameter that takes into account the deformability of the soil should also be included. The expressions of the CRR and the CSR are shown below.

Formula provided by Boulanger and Idriss in 2014:

$${CRR}_{M=7.5,\sigma_{v}^{\prime }=1atm}=exp\left(\frac{{\left(N_{1}\right)}_{60cs}}{14.1}+{\left(\frac{{\left(N_{1}\right)}_{60cs}}{126}\right)}^{2}+{\left(\frac{{\left(N_{1}\right)}_{60cs}}{23.6}\right)}^{3}+{\left(\frac{{\left(N_{1}\right)}_{60cs}}{25.4}\right)}^{4}-2.8\right)$$ (1)

The resistance obtained thereby is valid for earthquakes with magnitudes of approximately 7.5 and an effective vertical stress of 1 atmosphere. Therefore, to introduce the characteristics of the earthquake and the depth of the soil element in the calculations, the following modifications are made in the CRR formula:

- Correction for depth, *K*_*σ*_:
  $$K_{\sigma }=1-C_{\sigma }\cdot Ln\left(\frac{\sigma_{v}^{\prime }}{P_{a}}\right)\leq 1.1$$ (2)
  where:
  $$C_{\sigma }=\frac{1}{18.9-2.55\cdot \sqrt{{\left(N_{1}\right)}_{60cs}}}\leq 1.1$$ (3)
- Correction based on the seismic moment, MSF:
  $$MSF=1+({MSF}_{max}-1)\cdot (8.64\cdot exp(\frac{{-M}_{w}}{4})-1.325)$$ (4)
  where:
  $${MSF}_{max}=1.09+{\left(\frac{{\left(N_{1}\right)}_{60cs}}{31.5}\right)}^{2}\leq 2.2$$ (5)

The formula to apply both corrections is as follows:

$${CRR}_{M,\sigma_{v}^{\prime }}={CRR}_{M=7.5,\sigma_{v}^{\prime }=1atm}\cdot MSF\cdot K_{\sigma }$$ (6)

Once the resistance of the soil element is calculated, the shear stress exerted on the soil element must be obtained using the next formula [3]:

$${CSR}_{M,\sigma_{v}^{\prime }}=0.65\cdot \frac{\sigma_{v}}{\sigma_{v}^{\prime }}\cdot \frac{a_{max}}{g}\cdot r_{d}$$ (7)

*r*_*d*_ is the coefficient of the shear stress reduction, the arguments of which appear inside the parentheses (in radians).

$$r_{d}=exp(\alpha +\beta \cdot M_{w})$$ (8)

The following coefficients, *α* and *β*, are the adjustment values and are dependent on the depth, *z*, below the surface of the soil (in meters):

$$\alpha =-1.012-1.126\cdot sin\left(\frac{z}{11.73}+5.133\right)$$ (9)

$$\beta =0.106+0.118\cdot sin(\frac{z}{11.28}+5.133)$$ (10)

In conclusion, to obtain the FSL:

$$FSL=\frac{{CRR}_{M,\sigma_{v}^{\prime }}}{{CSR}_{M,\sigma_{v}^{\prime }}}$$ (11)

**Fig 2:**
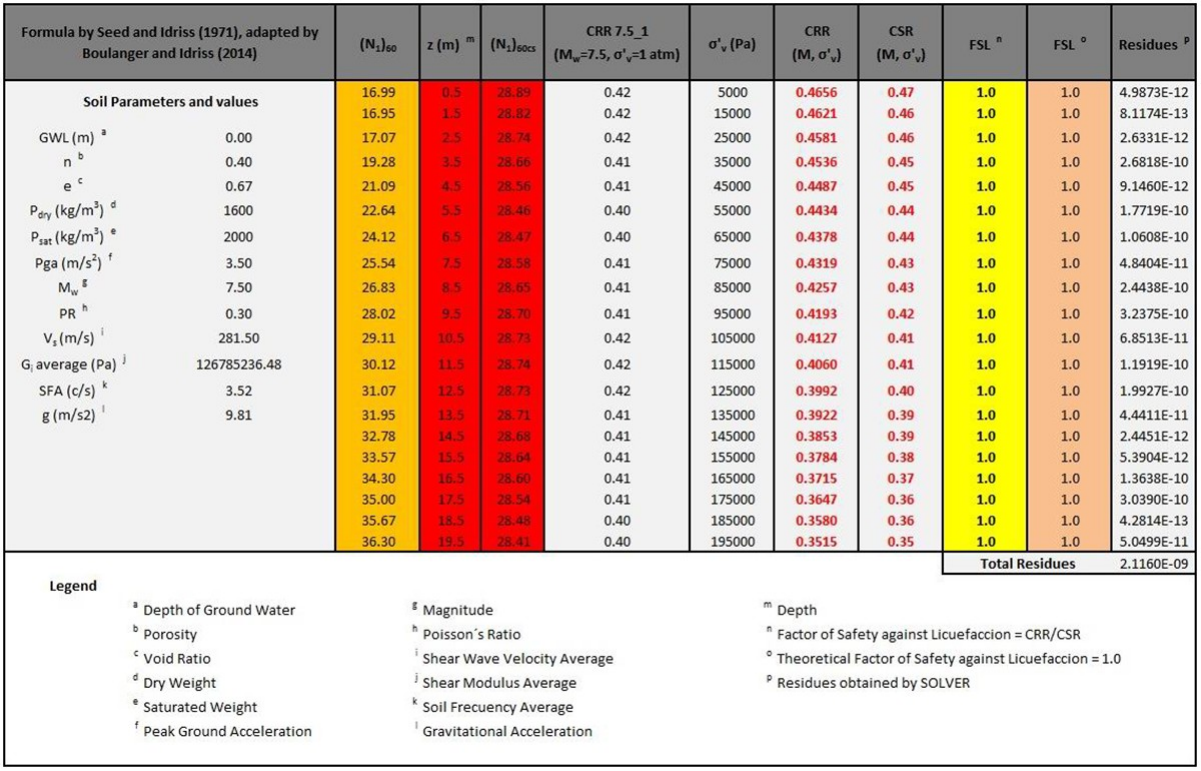
Excerpt of the spreadsheet used to obtain the *(N*_*1*_*)*_*60cs*_ value verifying that FSL = 1, according to the Seed and Idriss model (1971), its formula adapted by Boulanger and Idriss (2014) for the Puerto Quellon (PQ) accelerogram case.

By means of the previous expressions, it is possible to determine the *(N*_*1*_*)*_*60cs*_ values to verify FSL = 1 for each of the earthquakes listed in Table 3 and for different depths (from 0 to 20 m). For this purpose, we use a spreadsheet and a tool (SOLVER) to minimize the sum of residuals, defined as *(FSL-1)*^*2*^, by changing the cells of *(N*_*1*_*)*_*60*_. In Fig 2, an example is shown for the Puerto Quellon (PQ) case.

The results shown in the following Table 4 are obtained by carrying out the calculation of the *(N*_*1*_*)*_*60cs*_ values for all the seismic inputs and all the depths considered.

**Table 4:** *(N*_*1*_*)*_*60cs*_ values with FSL = 1 for each seismic input.

(N_1_)_60cs_ Values									
*z (m)*	1	2	3	4	5	6	7	8	9
	Central Italy (CI)	San Fernando (SF)	Imperial Valley (IV)	Tabas Iran (TI)	Kern County (KC)	Puerto Quellon (PQ)	Maule (M)	Coquimbo (CO)	Coquimbo (CP)
**0.5**	12.4	20.1	30.5	7.6	18.6	28.9	20.1	31.0	35.1
**1.5**	12.3	20.0	30.4	7.5	18.4	28.8	20.1	30.9	35.1
**2.5**	12.0	19.8	30.3	7.3	18.3	28.7	20.1	30.9	35.1
**3.5**	11.8	19.5	30.2	7.3	18.1	28.7	20.1	30.9	35.1
**4.5**	11.8	19.3	30.1	7.4	17.9	28.6	20.0	30.8	35.1
**5.5**	11.8	19.3	30.0	7.5	18.0	28.5	20.4	30.8	35.0
**6.5**	11.8	19.3	29.9	7.5	18.1	28.5	20.8	30.8	35.0
**7.5**	11.6	19.2	29.9	7.4	18.2	28.6	21.2	30.9	35.1
**8.5**	11.4	19.1	29.9	7.3	18.1	28.6	21.6	31.0	35.1
**9.5**	11.3	19.0	29.9	7.2	18.1	28.7	21.9	31.2	35.3
**10.5**	11.0	18.8	29.9	7.1	18.0	28.7	22.1	31.3	35.4
**11.5**	10.8	18.6	29.8	6.9	17.9	28.7	22.3	31.4	35.4
**12.5**	10.5	18.4	29.7	6.7	17.7	28.7	22.5	31.5	35.5
**13.5**	10.2	18.1	29.7	6.5	17.6	28.7	22.7	31.6	35.6
**14.5**	9.9	17.8	29.6	6.3	17.4	28.7	22.9	31.6	35.6
**15.5**	9.5	17.5	29.5	6.1	17.2	28.6	23.0	31.7	35.7
**16.5**	9.2	17.2	29.3	5.9	17.0	28.6	23.1	31.7	35.7
**17.5**	8.9	16.9	29.2	5.6	16.7	28.5	23.1	31.8	35.8
**18.5**	8.5	16.6	29.1	5.4	16.5	28.5	23.3	31.8	35.8
**19.5**	8.2	16.2	29.9	5.2	16.2	28.4	23.3	31.8	35.8

### Numerical model in FLAC3D: Characteristics and properties

In this stage, the numerical model is defined, and its initial and boundary conditions, constitutive model and input properties are assigned.

**Fig 3:**
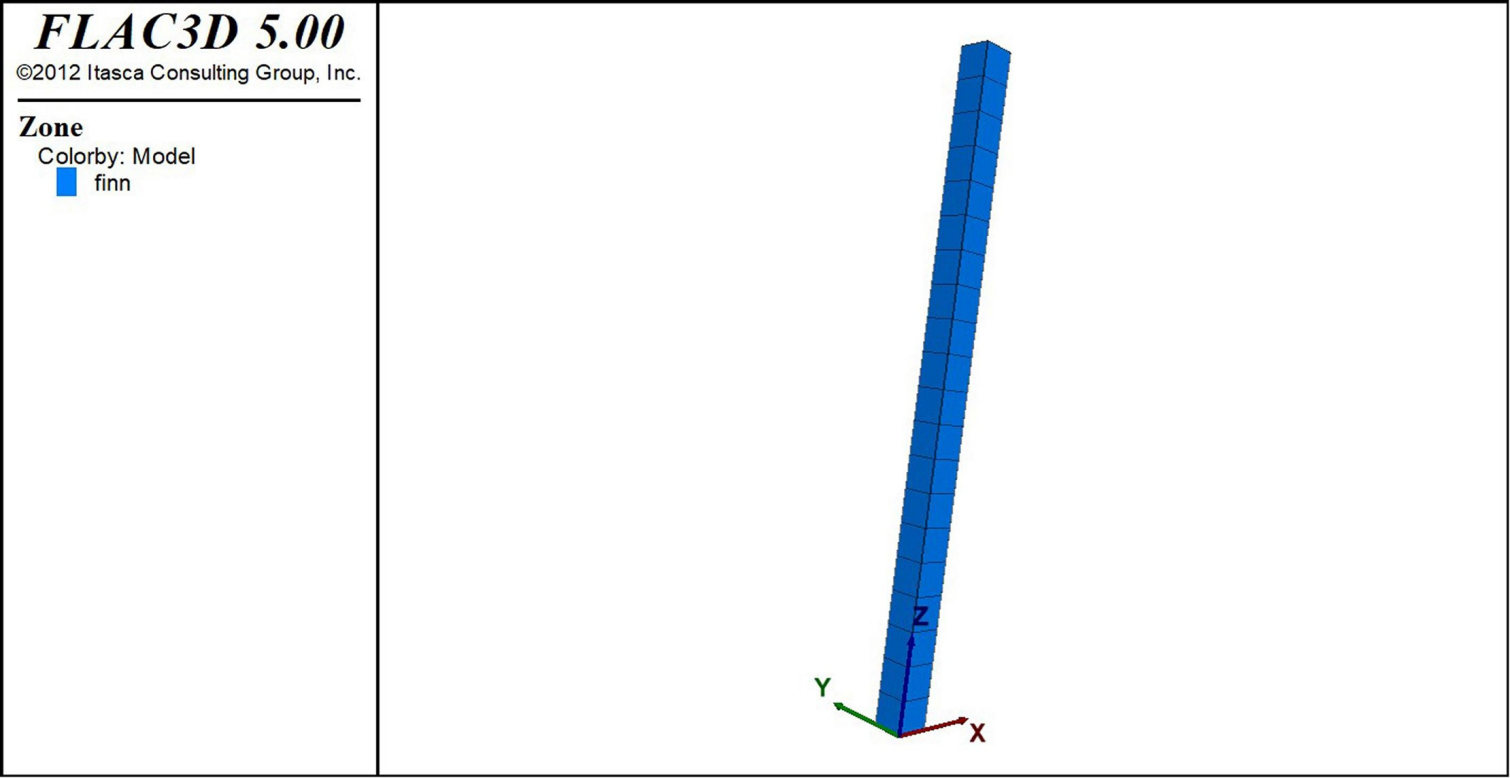
Geometry of the model designed for this study.

The selected model represents a 20 m column of granular soil saturated subjected to a seismic input at its base. For this purpose, a mesh, made of 20 elements with dimensions of 1 x 1 x 1 m^3^ is constructed in FLAC3D, as shown in Fig 3.

Periodic conditions linking the nodes on one side of the model to those on the opposite side are used as boundary conditions to induce identical behavior. The lateral confinement that cannot be implemented through the anchorage of the lateral displacements in this dynamic model is thus represented.

The initial stress conditions are due to the existing overburden material load (lithostatic); hence, an at-rest thrust coefficient of *k*_*0*_ *= 1*, which is considered to adequately represent the stress state of incompetent grounds, is used. In geological time periods, this coefficient tend to dissipate the deviator stresses and therefore equalize the vertical and horizontal stresses (Heim’s rule).

The Finn constitutive model incorporates the Byrne equation [1] for pore pressure generation due to cyclic strain. This constitutive model, proposed by Martin et al. [23], is a classic model with a Mohr-Coulomb failure criterion that, via an internal algorithm, tests the load-unload-reload cycles for each element in the model and determines the cyclic strain generated by each cycle. Then, the algorithm calculates the increase in volumetric strain associated with the cycle and the shear stress using the following expression:

$${\left({\Delta \varepsilon }_{v}\right)}_{\frac{1}{2}cycle}=\gamma_{c}C_{1}exp(-C_{2}\frac{\varepsilon_{vd}}{\gamma_{c}})$$ (12)

where Δ*ε*_*v*_ is the variation in the volumetric strain experienced by a soil element mid-cycle (load and unload), *ε*_*v*_ is the accumulated volumetric strain up to the previous cycle, *γ*_*c*_ is the cyclic shear strain, and *C*_*i*_ are constants that are dependent on the *(N*_*1*_*)*_*60cs*_ value.

$$C_{1}^{c}=8.7{\left(N_{1}\right)}_{60cs}^{-1.25}$$ (13)

$$C_{1}=0.5C_{1}^{c}$$ (14)

$$C_{2}=0.4/C_{1}^{c}$$ (15)

Lastly, the model generates an increase in pore pressure for each cycle considered; this increase is obtained by multiplying the volume increase associated with the cycle, provided by the equation of the volumetric strain and by the soil bulk modulus.

Given that the Finn model includes a classic Mohr-Coulomb failure criterion, two elastic and three plastic properties are required. For this study, pore pressure generation is analyzed in more detail. Thus, the plastic properties are not very relevant because the model is developed by assuming an elastic case. Therefore, a high value is assigned to avoid breakage of the soil and destabilization in the calculation of the model.

With regard to the elastic properties and given that the relevant variable is the *(N*_*1*_*)*_*60cs*_ value, the initial shear modulus *(G*_*i*_*)* is calculated, in *Pa*, using the following elastic relationship:

$$G_{i}=\rho_{sat}\cdot V_{s}^{2}$$ (16)

where *ρ*_*sat*_ is the saturated density of the soil, in kg/m^3^, and *V*_*s*_ is the shear wave velocity in the soil, in m/s, which depends on the *(N*_*1*_*)*_*60cs*_ value via the following expression [32]:

$$V_{s}=98.1\cdot {\left({\left(N1\right)}_{60cs}\right)}^{0.32}$$ (17)

Therefore, drawing on both formulas, the initial shear modulus is:

$$G_{i}=\rho_{sat}\cdot 9264\cdot {\left({\left(N1\right)}_{60cs}\right)}^{0.64}$$ (18)

The properties assigned to the Finn model incorporated in FLAC3D are listed in, Tables 5, 6 and 7.

**Table 5:** Specific elastic properties introduced into the Finn model.

Finn model. Elastic Parameters			
G_i_ (Pa)	Poisson’s Ratio	ρ_d_ (kg/m^3^)	ρ_sat_ (kg/m^3^)
Values obtained by Equation (18)	0.3	1,600	2,000

**Table 6:** Specific plastic properties introduced into the Finn model.

Finn model. Plastic Parameters			
c (kPa)	ϕ (o)	Traction (kPa)	Dilatancy (ρ)
High values to avoid soil breakage: 1e^20^	0	High values to avoid soil breakage: 1e^20^	0

**Table 7:** Other specific parameters introduced into the Finn model.

Finn model. Other Parameters	
(N_1_)_60cs_	*ff_latency*
Variable with the earthquake moment magnitude and depth	50

The *ff_latency* parameter is employed in FLAC3D to delineate the number of steps in the calculation. This internal parameter remains defined as the minimum number of time steps between the stress reversals, [33]. *ff_latency* is a control parameter used to stabilize the model and help with convergence, serving as a filter so that there are not too many oscillations during modeling.

The previously identified properties refer to the static model; thus, in addition to these properties, the dynamic behavior of the soil must be considered. This behavior is modeled by adopting one of the hysteretic models implemented in FLAC3D, specifically, the sigmoidal model Sig3. The equation of the aforementioned model is displayed below, and the parameters listed in Table 8.

$$\frac{G}{G_{i}}=\frac{a}{1+exp(\frac{-({log}_{10}(\gamma_{c})-x_{0})}{b})}$$ (19)

**Table 8:** Parameters of the sigmoidal hysteretic model Sig3.

Sigmoidal Hysteretic model Sig3 in FLAC3D. Specific Values		
a	b	X_0_
1	-0.5	-0.7

**Fig 4:**
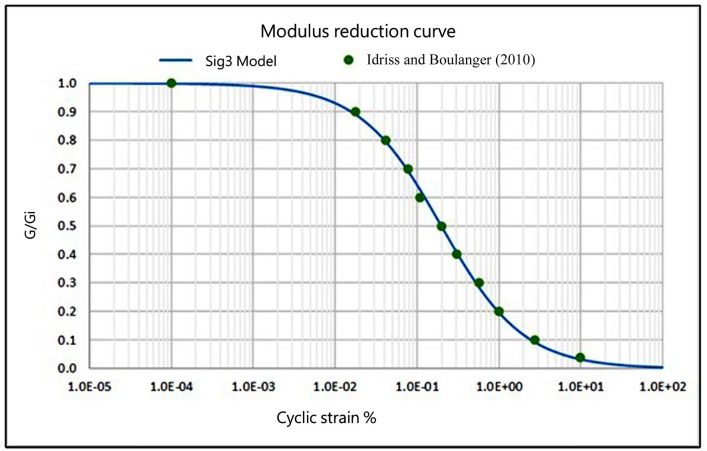
Shear modulus reduction curve.

The reduction curve of the shear modulus generated by these parameters, shown in Fig 4, is selected after various tests, based on those used by Idriss and Boulanger [16], to obtain the reduction factor of the shear stress by depth (*r*_*d*_), to find which value provides the best results.

Once the properties of the mechanical model are defined, the configuration of the flow model settings must be described. In this case, a model with hydromechanical coupling and impeded flow is used; in other words, the generated volume undergoes variations that the ground cannot dissipate. This hypothesis is justified given that in the brief time lapse of the duration of the seismic stimulation, the produced flow could be considered negligible even if the permeability of the soil is of an average type. Shown in Table 9.

**Table 9:** Parameters of the flow model.

Flow model in FLAC3D. Properties				
Coupling	K (m/s)	n	K_w_ (Pa)	Cavitation (Pa)
Hydromechanical with impeded flow	1e^-4^	0.4	2.2e^9^	High values to avoid instabilities: -1e^5^

## Results

This section presents the results obtained in the verification process of the equation of the pore pressure generation, Byrne [1]. The stimulated numerical model is run with each of the seismic inputs displayed in Table 3 applied to its base, and the results are expressed in terms of the FSL for each soil element, defined as

$$FSL=\frac{\sigma_{m}^{\prime }(t_{o}=0)}{pp(t_{f}-t_{o})}$$ (20)

It is well known that this definition of the FSLs diverges from that recommended by Seed and Idriss in their proposal. However, it is widely held that these two expressions must be equivalent at FSL = 1. Therefore, to verify the usefulness of Byrne’s equation to generate excess pore pressure until reaching the liquefaction of the ground, this assumption is reasonable.

If the Byrne model were to yield the expected results, the obtained FSL should be equal to unity. However, it can be expected that, given that the verification equation is not an analytical expression with boundary conditions and specific initial inputs or with a totally defined dynamic load, differences arise between the empirical and numerical results. Hence, analytical studies such as this work are needed. In this last case, considering the results provided by the Seed and Idriss model (1971), which was adapted by Boulanger and Idriss in 2014, we will proceed to modify the parameters of the Byrne equation to achieve good compatibility between these two formulas for FSL = 1. In addition, a convenient modification for FSL = 1.3 will be researched.

To analyze the suitability of the FSL vs. depth results, an analysis of the data by using basic statistical variables (average, standard deviation, and coefficient of variation) is performed.

### Initial FSL (precorrection)

To resolve each of the cases, the process begins by statistically balancing the model and storing the effective mean stress and the pore pressure of each element in one memory position. Then, the corresponding *(N*_*1*_*)*_*60cs*_ value is assigned according to the element depth (see example Table 4) and the features of the earthquake, namely, *M*_*w*_ and *Pga* (see Table 3). Finally, the seismic input is applied to the base of the soil column from *t = t*_*0*_ to *t = t*_*f*_.

During the development of the earthquake, the pore pressure excess is tested, measured as the difference between the pressure at time *t* and the previously stored pressure at time *t*_*0*_; with this detail and the effective mean pressure, also measured and stored, the evolution of the FSL is continuously monitored.

**Fig 5:**
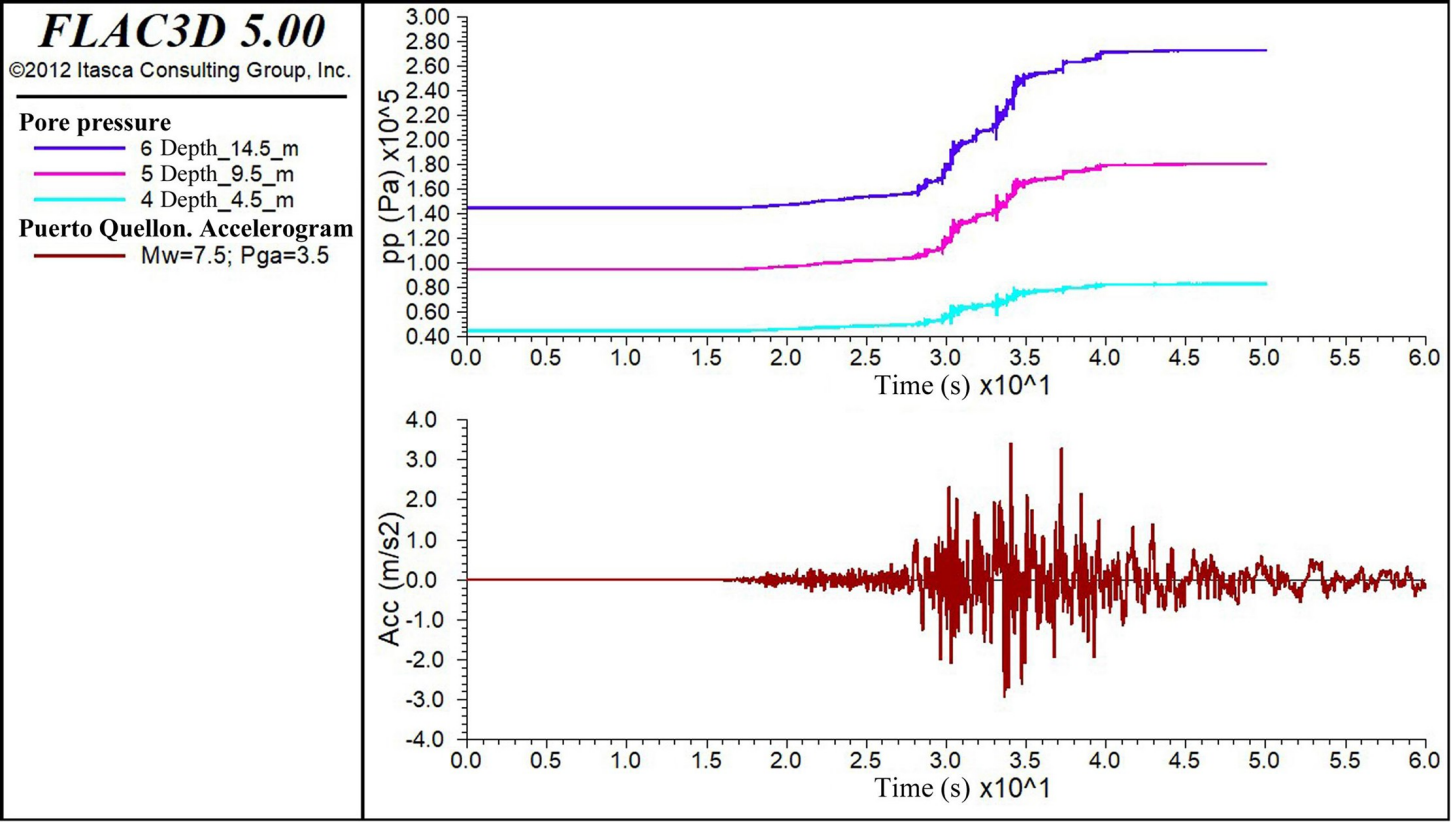
Evolution of the pore pressures vs. input accelerogram for Puerto Quellon (PQ).

Displayed (Fig 5) below is the output of the model in terms of the evolution of the pore pressure for three depths: 4.5, 9.5 and 14.5 m. This graph supplements the accelerogram of the prescribed earthquake.

In Fig 5, it is possible to confirm when the increase in the pore pressure undergoes its greatest change with the peak ground acceleration; however, after this, the majority of excess pore pressure generated during strong shaking is (generally) low, except when there are acceleration peaks similar to the Pga in question at a later stage.

**Fig 6:**
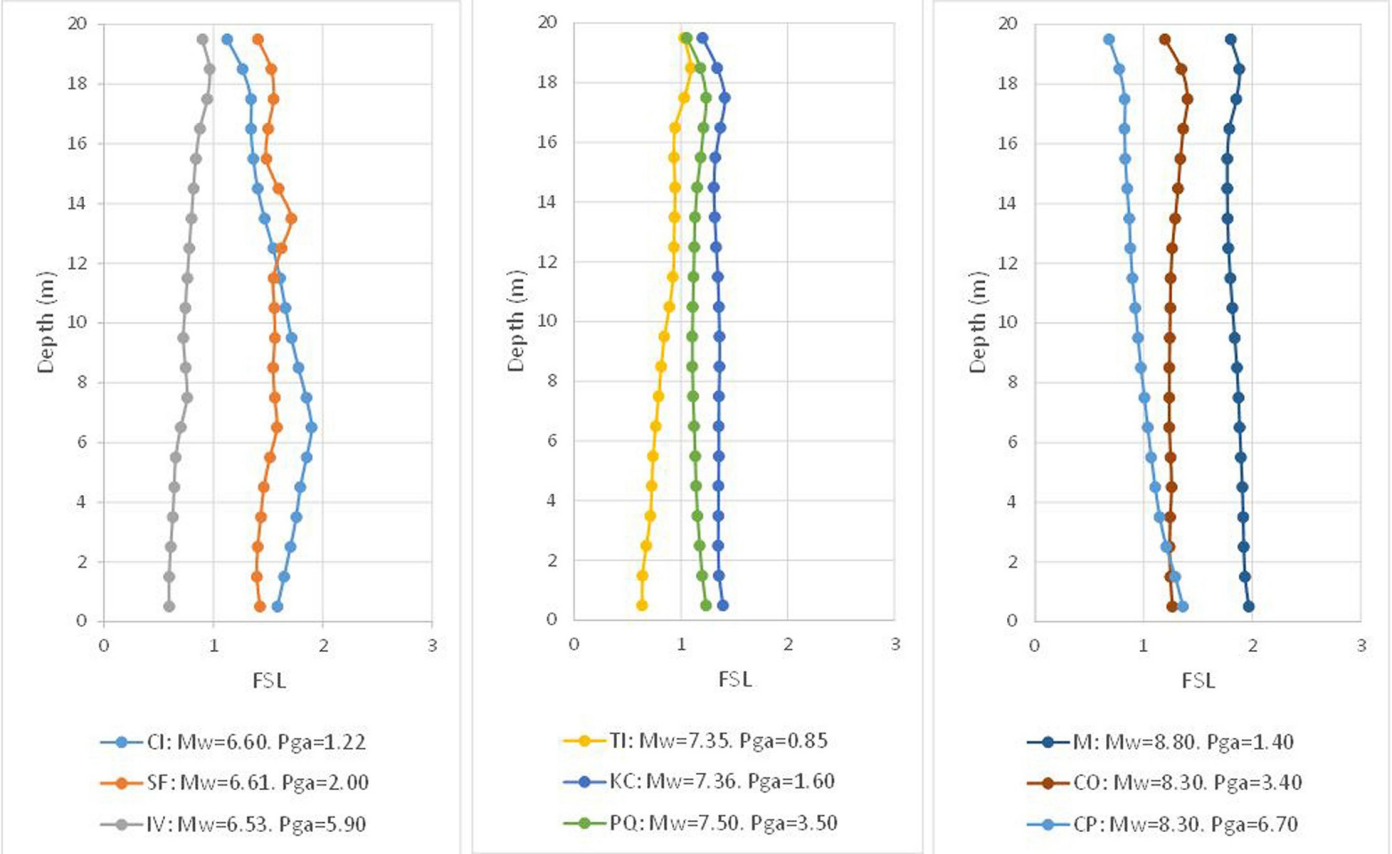
Numerical factor of safety vs. depth using the *(N*_*1*_*)*_*60cs*_ value verifying FSL = 1, according to the Seed and Idriss model (1971) adapted by Boulanger and Idriss (2014).

In Fig 6, the results of the FSL are provided for each range of *M*_*w*_ and each depth. Finally, it is possible to verify the degree of closeness between the Byrne model (incorporated in FLAC3D) and that of Seed and Idriss, whose formula was adapted by Boulanger and Idriss.

**Fig 7:**
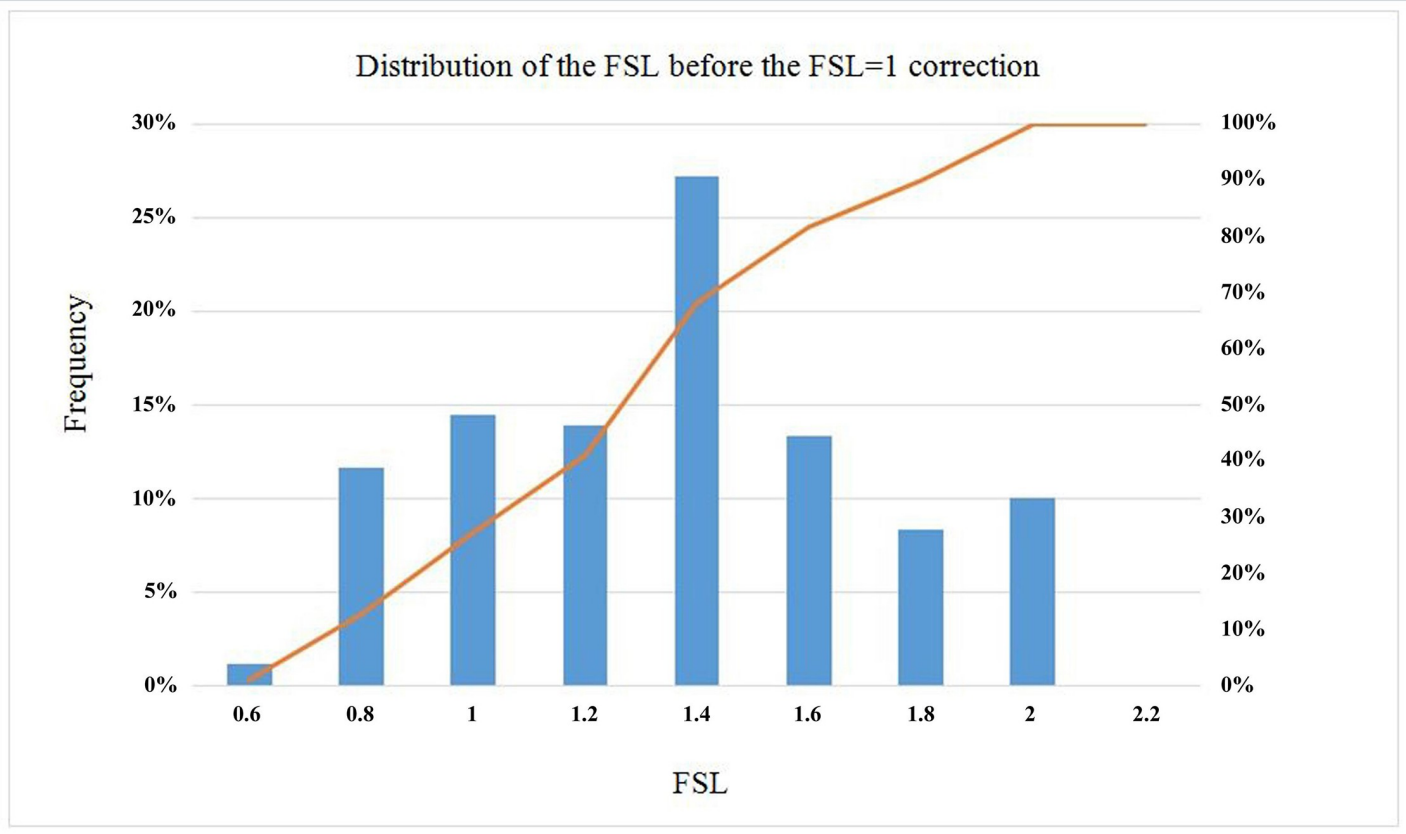
Distribution of the numerical factor of safety before correction.

It is possible to observe that the obtained result is acceptable, even if cases appear such as that of Imperial Valley (IV) and Coquimbo (CP), which provide an FSL that is sensibly lower than unity. Similarly, the opposite case is shown for Maule (M), which presents a notably higher FSL. For an overview of the obtained results, these cases are grouped together in the histogram of Fig 7.

The distribution of the FSL displayed in the previous histogram clearly reflects a high degree of dispersion and a bias in underestimating the risk of liquefaction with respect to the Seed and Idriss formula; the statistics of the results are displayed below in Table 10.

**Table 10:** Statistical results of the FSLs obtained with the Byrne equation (FLAC3D) for FSL = 1 according to the Seed and Idriss model (1971) adapted by Boulanger and Idriss (2014).

Statistical Values of the FSL Result			
Average	Standard Deviation	Coefficient of Variation (%)	% > 1.3
1.26	0.36	28.78	≈50

The table highlights the percentage of the FSL results greater than 1.3, given that FSL>1.3 is generally required for it to be accepted as a reliable prediction of the risk of liquefaction.

#### Correction of the Byrne equation (1991)

In view of the above results, the authors consider it necessary to revise the parameters of the Byrne equation to better adjust the Seed and Idriss method adapted by Boulanger and Idriss, which is postulated (by consensus) to be correct.

**Fig 8:**
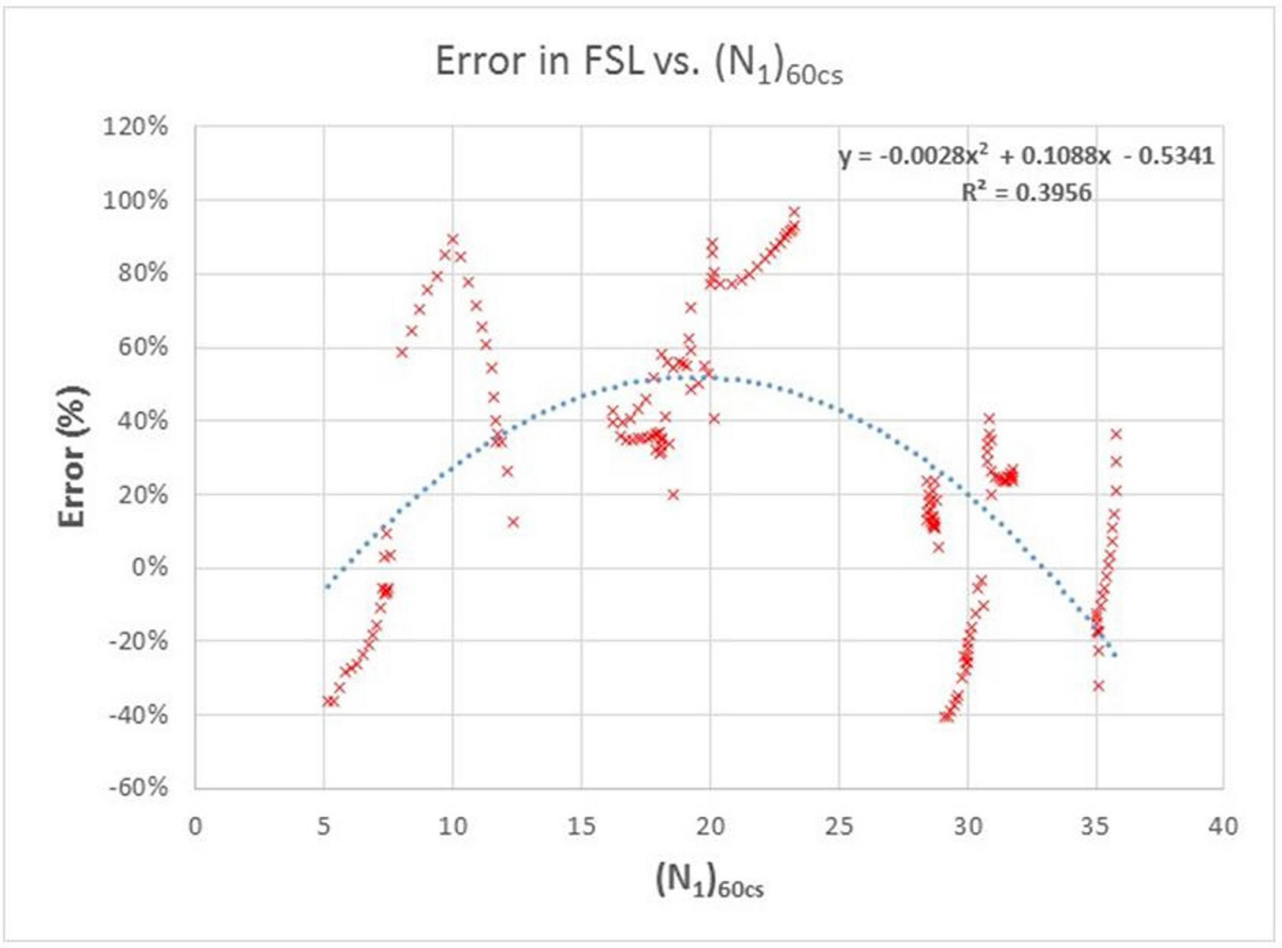
Percentage error of the numerical model against the solution obtained with the method of Seed and Idriss (1971) adapted by Boulanger and Idriss (2014).

In the first instance, the error with respect to FSL = 1 vs. *(N*_*1*_*)*_*60cs*_ is plotted to verify some type of relationship, as shown in Fig 8.

The previous figure clearly suggests the possibility of acting on the same *(N*_*1*_*)*_*60cs*_ value so that the correction would be scarce or null for the high and low values and high for the average values.

To carry out this modification of (N_1_)_60cs_, a statistical analysis of the data obtained via linear approximation is carried out to improve the results, validating the proposal.

Proceed as follows: the original value of *(N*_*1*_*)*_*60cs*_, provided by the model of Seed and Idriss adapted by Boulanger and Idriss, will be tentatively corrected with a linear adjustment, depending on the error, so that a new test value, i.e., *(N*_*1*_*)*_*60cs_corr*_ will be obtained. This correction will be carried out for each seismic input of the nine selected cases.

$${\left(N_{1}\right)}_{60cs\_corr}={\left(N_{1}\right)}_{60cs}+{\left(N_{1}\right)}_{60cs}\cdot a_{i}*(FSL-1)$$ (21)

Once the first attempt is carried out, the nine cases are recalculated in FLAC3D, and their approximation to FSL = 1 is verified; the value provided for the previous equation is *a*_*i*_ *= -0*.*5*. Clearly, some cases will be corrected to a greater degree than others; thus, in the following attempt, only those that did not converge to FSL = 1 are corrected.

**Fig 9:**
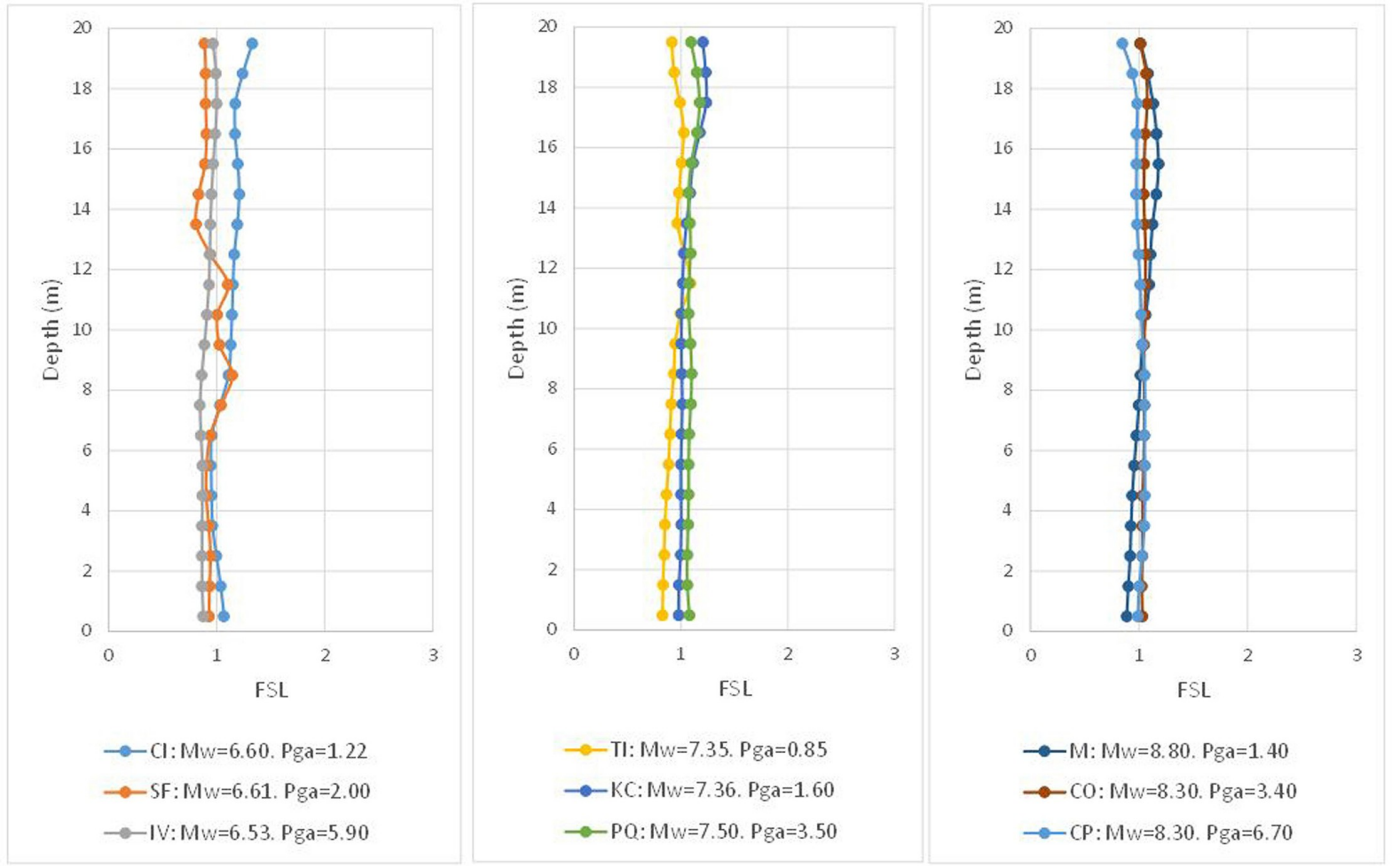
Numerical factor of safety vs. depth using *(N*_*1*_*)*_*60cs_corr*_ to verify FSL = 1, according to the Seed and Idriss model (1971) adapted by Boulanger and Idriss (2014).

The results of this approximation process, i.e., *(N*_*1*_*)*_*60cs_corr*,_ which was introduced in the Byrne equation, verify the Seed and Idriss model, as shown in Table 11, and the result of the adjustment in terms of the factor of safety is shown in Fig 9.

**Table 11:** The obtained *(N*_*1*_*)*_*60cs_corr*_ for FSL = 1 for each seismic input.

(N_1_)_60cs_corr_ Values									
z (m)	Central Italy (CI)	San Fernando (SF)	Imperial Valley (IV)	Tabas Iran (TI)	Kern County (KC)	Puerto Quellon (PQ)	Maule (M)	Coquimbo (CO)	Coquimbo (CP)
**0.5**	9.86	12.83	35.39	8.22	15.02	28.08	13.85	27.89	40.73
**1.5**	8.97	11.75	34.18	7.84	13.76	26.19	12.92	25.52	39.06
**2.5**	8.42	11.47	34.44	7.93	13.05	25.35	13.23	24.64	38.15
**3.5**	8.26	11.72	35.45	8.26	13.25	25.61	13.97	25.21	38.16
**4.5**	8.14	11.69	35.92	8.44	13.47	25.91	14.16	25.59	38.02
**5.5**	7.94	10.85	36.11	8.44	13.71	26.30	14.41	25.89	37.68
**6.5**	7.58	9.94	36.34	8.45	13.71	26.59	14.69	26.29	37.31
**7.5**	7.11	10.60	36.69	8.42	13.63	26.79	14.86	26.87	37.20
**8.5**	6.71	11.10	37.00	8.35	13.50	26.95	14.89	27.22	36.99
**9.5**	6.35	10.96	37.27	8.36	13.39	27.09	14.84	27.39	36.62
**10.5**	5.96	10.82	37.52	8.38	13.27	27.18	14.75	27.55	36.29
**11.5**	5.53	10.82	37.12	8.30	13.18	27.19	14.64	27.69	35.87
**12.5**	5.06	10.57	36.81	8.17	13.13	27.10	14.59	27.77	35.38
**13.5**	4.71	10.28	37.66	8.02	13.02	26.93	14.56	27.83	34.90
**14.5**	4.75	10.57	38.30	7.85	12,87	26.76	14.50	27.70	34.38
**15.5**	4.81	10.80	38.38	7.62	12.74	26.61	14.42	27.60	33.76
**16.5**	4.78	10.78	38.46	7.38	12.59	26.37	14.39	27.83	33.07
**17.5**	4.80	10.79	38.62	7.20	12.43	26.02	14.36	27.96	32.02
**18.5**	4.83	10.63	38.69	7.01	12.19	25.63	14.26	27.87	30.56
**19.5**	4.84	10.23	38.53	6.71	11.73	25.06	13.81	27.57	29.31

**Fig 10:**
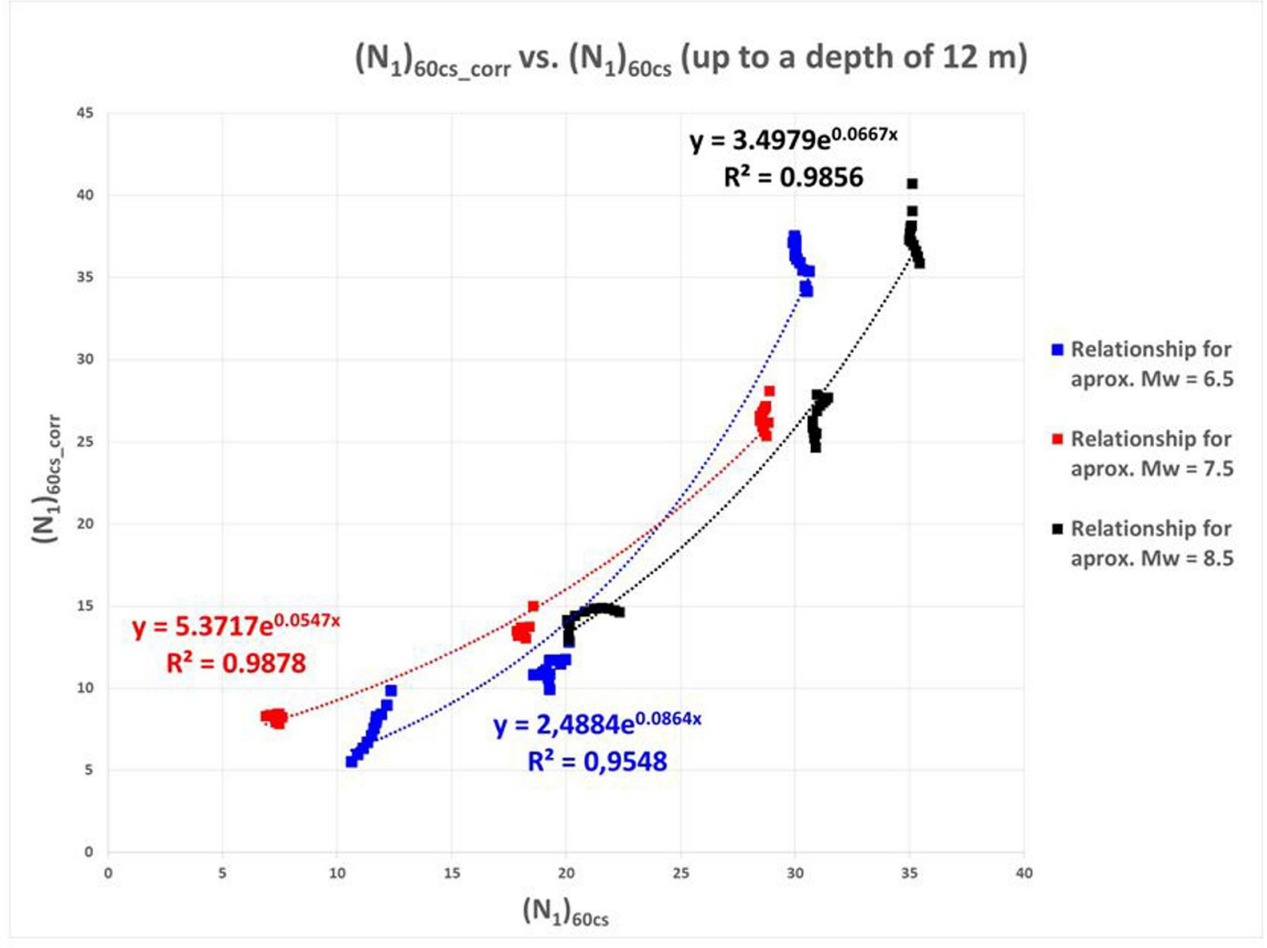
Relationship between (N_1_)_60cs_corr_ vs. (N1)_60cs_ obtained for the nine cases considered.

With regard to Fig 9, the result is deemed adequate, and the *(N*_*1*_*)*_*60cs_corr*_ value is compared with that of *(N*_*1*_*)*_*60cs*_, as shown Fig 10; however, the data for depths under 12 m will not be taken into account to achieve a better adjustment because, at greater depths, there is increased dispersion in the *r*_*d*_ factor of the Seed and Idriss model adapted by Boulanger and Idriss. Beyond that depth, *r*_*d*_ is significantly affected by the frequency content of the seismic signal as Ishihara [34] and Golesorkhi [8] mentioned and collected by Idriss and Boulanger [16].

In the previous figure, the exponential relationship between (N_1_)_60cs_ and (N_1_)_60cs_corr_ can be clearly seen. By exploiting this trend, it is possible to determine the values for the earthquake magnitude, M_w_, by establishing three correlations, for M_w_ values of 6.5, 7.5 and 8.5.

Therefore four relationships are proposed, as listed below, to allow a shift from *(N*_*1*_*)*_*60cs*_ to *(N*_*1*_*)*_*60cs_corr*_:

$${\left(N_{1}\right)}_{60cs\_corr}\approx a_{1}\cdot exp(a_{2}\cdot {\left(N_{1}\right)}_{60cs})$$ (22)

where:

$$a_{1}=\left\{ \begin{array}{l} 4.1438\to Global\;Case\\ 2.4884\to \;M_{w}\approx 6.5\\ 5.3717\to \;M_{w}\approx 7.5\\ 3.4979\to \;M_{w}\approx 8.5 \end{array}\right.\;a_{2}=\left\{ \begin{array}{l} 0.0632\to Global\;Case\\ 0.0864\to \;M_{w}\approx 6.5\\ 0.0547\to \;M_{w}\approx 7.5\\ 0.0667\to \;M_{w}\approx 8.5 \end{array}\right.$$ (23)

This allows us to obtain the *(N*_*1*_*)*_*60cs_corr*_ value and serves as a new entry in the Byrne equation. In this way, the specific coefficients of each moment magnitude range can be used.

### Final FSL (postcorrection)

**Fig 11:**
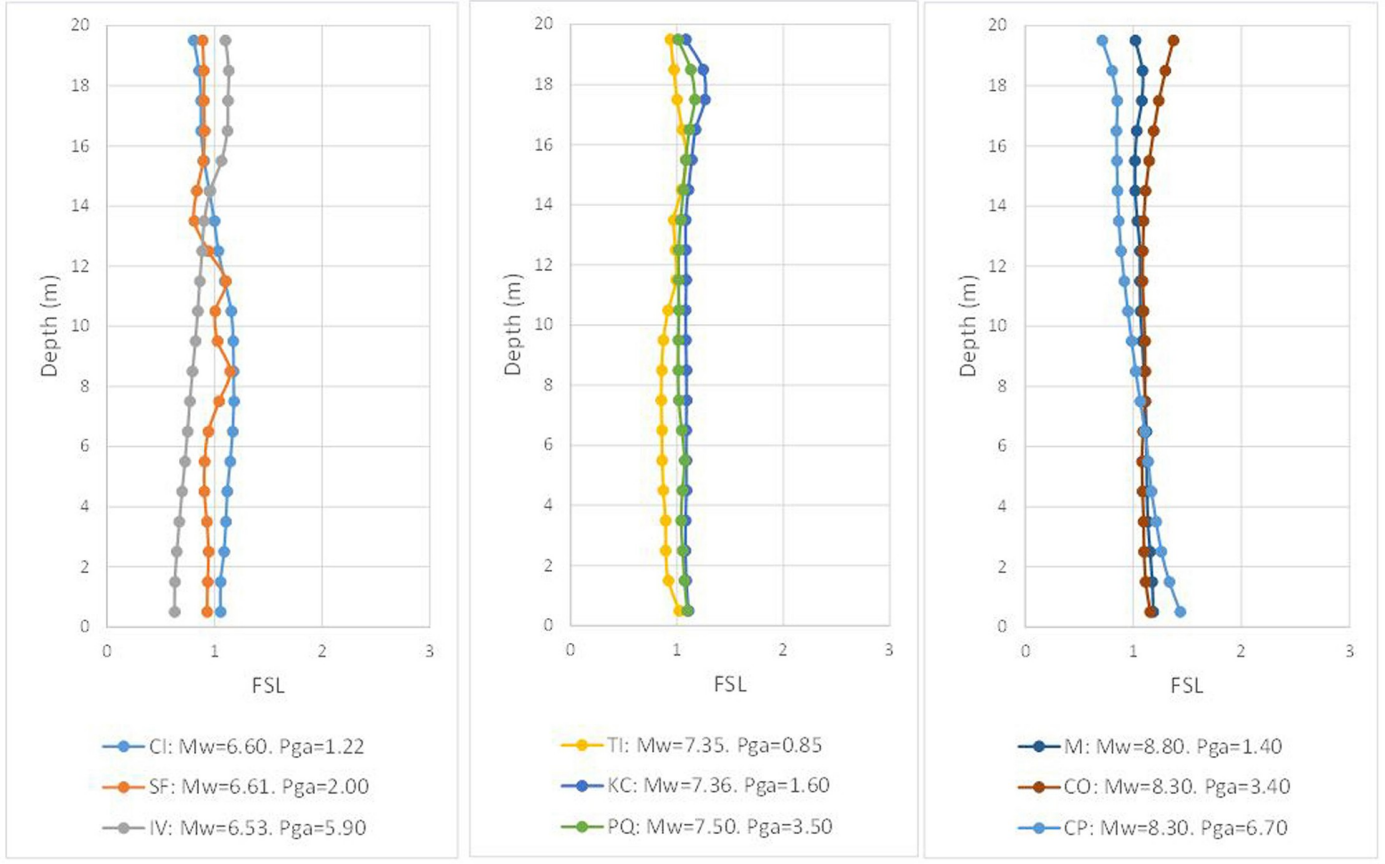
Numerical factor of safety vs. depth using *(N*_*1*_*)*_*60cs_corr*_ to verify FSL = 1, according to the Seed and Idriss model (1971) adapted by Boulanger and Idriss (2014).

The next step is to verify the results with the proposed modification for the *(N*_*1*_*)*_*60cs*_ value. The first step consists of correcting values of Table 4 according to the previous equation, with the adequate coefficients, and assigning them to the elements of the numerical model. Then, the FLAC3D model is developed with the nine seismic hypotheses, and the results are plotted for each earthquake in terms of the FSL and depth at the end of the dynamic action in Fig 11.

**Fig 12:**
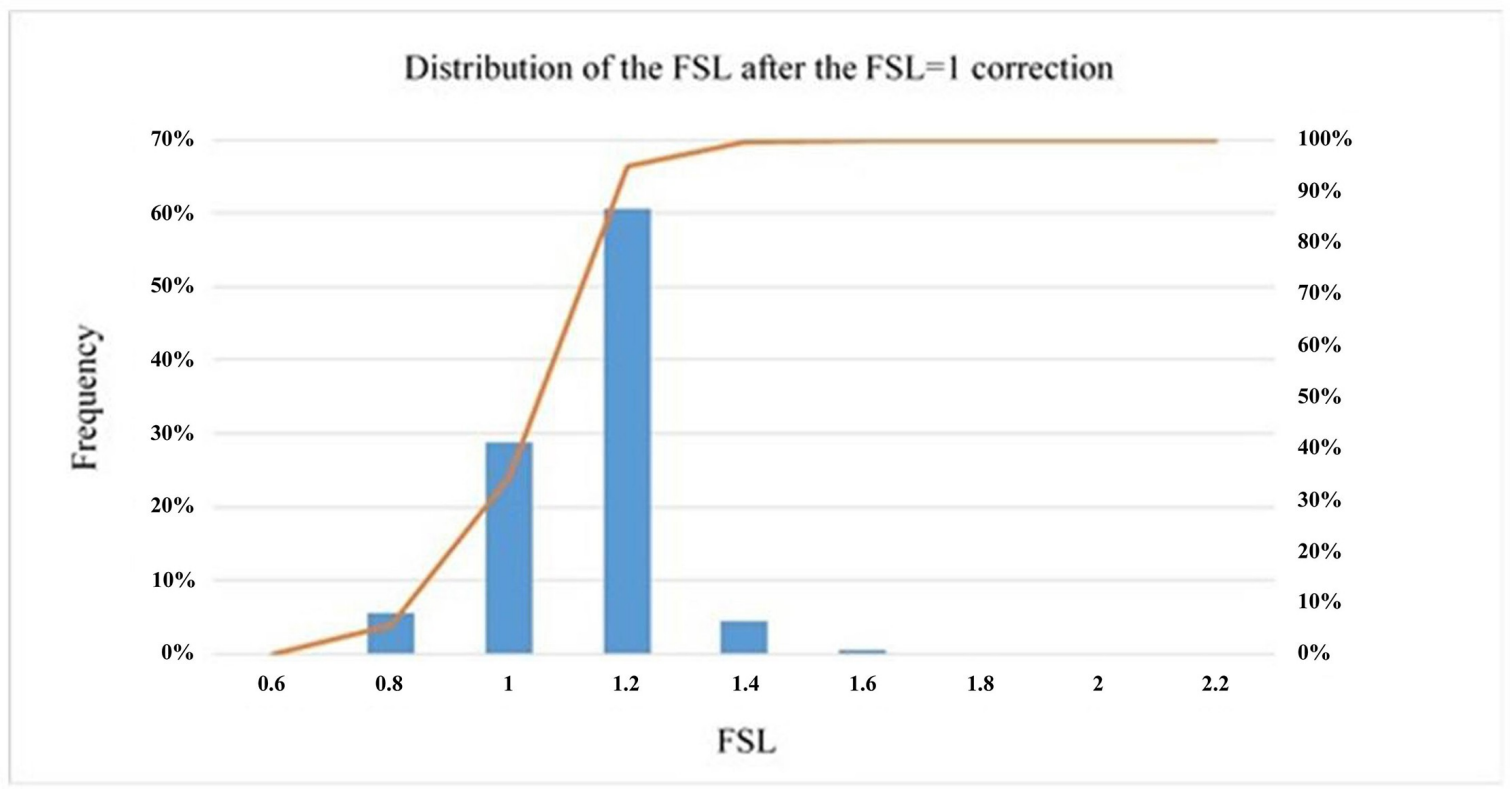
Distribution of the numerical factor of safety after correction.

Visual inspection of the previous figure suggests that the correction carried out via the proposed correlation for four magnitudes M_w_ improves the result the Byrne equation produces in the numerical model. However, for the precorrection hypotheses, the histogram of values is presented in Fig 12.

The factor of safety distribution is tightly concentrated around FSL = 1, although a percentage of cases persist that undervalue the resistance to liquefaction. Table 12 shows the statistical values of the previous distribution.

**Table 12:** Statistical results when the FSL is obtained with the corrected Byrne equation (FLAC3D) for FSL = 1 according to the Seed and Idriss model (1971) adapted by Boulanger and Idriss (2014).

Statistical Values of the FSL Result			
Average	Standard Deviation	Coefficient of Variation (%)	% > 1.3
1.02	0.14	13.58	≈5

Considering the numerical parameters that describe the distribution of the results, the improvement in the degree of dispersion is evident, as it decreases from 28.8% to 13.6%. Furthermore, only 5% of the cases present a factor of safety in excess of 1.3; consequently, overestimation of the resistance to liquefaction is limited to a few cases.

In addition to the verification of the case of FSL = 1, the calculations are repeated for FSL = 1.3 to verify that the results provided by the proposed correction are achievable for other ranges of the factor of safety. Therefore, first, in the spreadsheet prepared for this purpose, we obtain the *(N*_*1*_*)*_*60cs*_ value that verifies a factor of safety of 1.3 for all earthquakes and depths, which are then introduced into the FLAC3D numerical model for the nine tested cases.

**Fig 13:**
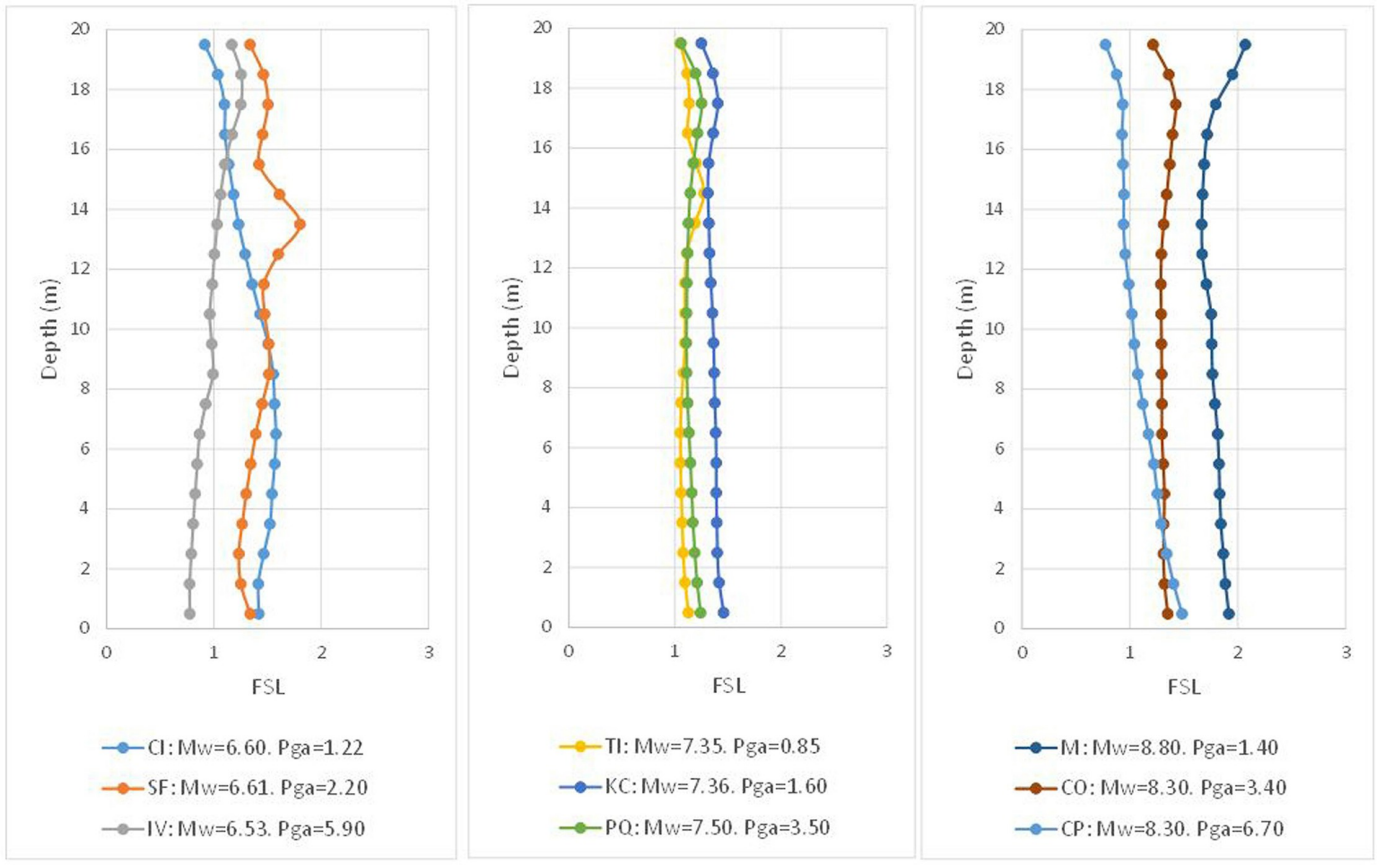
Numerical factor of safety vs. depth using the *(N*_*1*_*)*_*60cs_corr*_ value to verify FSL = 1.3 according to the Seed and Idriss model (1971) adapted by Boulanger and Idriss (2014).

**Fig 14:**
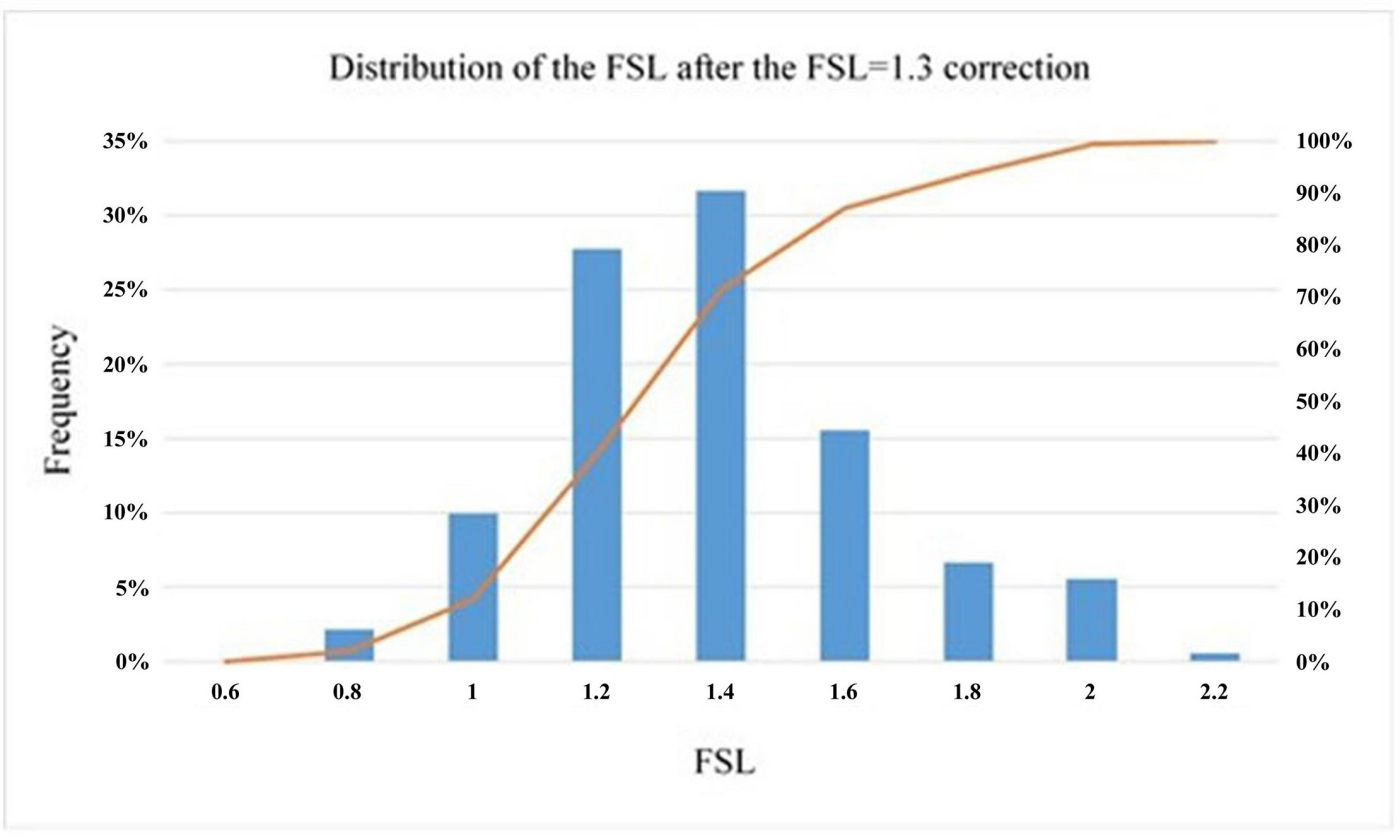
Distribution of the numerical factor of safety after the FSL = 1.3 correction.

The results of this last hypothesis are shown below in Figs. 13 and 14 and Table 13:

**Table 13:** Statistical results upon obtaining the FSL with the corrected Byrne equation (FLAC3D) for FSL = 1.3 according to the Seed and Idriss model (1971) adapted by Boulanger and Idriss (2014).

Statistical Values of the FSL Result			
Average	Standard Deviation	Coefficient of Variation (%)	% < 1
1.29	0.26	20.41	≈10

It is important to remember that the definition of the FSL proposed in this report and that used by Seed and Idriss [3] adapted by Boulanger and Idriss [17] are not the same and that they are only comparable in the case of FSL = 1. This discrepancy could cause the results obtained in this last hypothesis to diverge slightly from FSL = 1.3; nevertheless, as the distribution in Fig 14 and Table 13 show, only 10% of the cases are under 1.

## Conclusions

This study sought to verify the results of the Byrne equation [1], implemented in the Finn model by using FLAC3D, with respect to the generation of pore pressure in soils subjected to cyclic strain. The output results of the *(N*_*1*_*)*_*60*_ value of the SPT are compared with the FSL provided by the method proposed by Seed and Idriss [3] and adapted by Boulanger and Idriss [17]. The results are then introduced in the numerical model, with a purpose-built geometry, to which diverse seismic inputs are applied to its base. The FSL is defined as the ratio between effective mean stress and the pore pressure increase. Therefore, this factor of safety is obtained using the numerical model output values.

The factors of safety obtained by the numerical model using the selected *(N*_*1*_*)*_*60cs*_ value, which, according to the Seed and Idriss method adapted by Boulanger and Idriss, should be unitary, do approach this value. Nevertheless, the results display a certain dispersion and many cases present greater factors of safety (*FSL>1*.*3* ≈ *50%*), underestimating the risk of liquefaction.

A correction of the *(N*_*1*_*)*_*60cs*_ value is proposed according to equation (22) and its coefficients (23) based on *M*_*w*_, which allows us to obtain the *(N*_*1*_*)*_*60cs_corr*_ value and serves as a new entry in the Byrne equation.

After repeating the modeling by using the parameter of the corrected Byrne equation, a clear improvement is obtained in the output; the distribution of the factors of safety has a significantly reduced dispersion and fewer cases overestimate the risk of liquefaction, *FSL>1*.*3* ≈ *5%*.

Additionally, the calculation is repeated with the numerical model for a factor of safety of 1.3, verifying that the correction can obtain consistent FSLs, with a distribution whose average is 1.29 and that it provides only 10% false positives, namely, soil elements for which the numerical model determines them to be liquefied but the analysis does not.

Therefore, an equation is provided, serving as a correction of the Byrne equation for *(N*_*1*_*)*_*60*_ values of the normalized SPT test between 5 and 35 and soil depths to approximately 12 m in areas affected by earthquakes with magnitudes between 6.5 and 8.5.

## Supporting information

S1 File Supporting information. Statistical Data.
(PDF)
