# Supplementary material for: Adjustment of a numerical model for pore pressure generation during an earthquake
Source: PLoS One. 2019 Sep 26;14(9):e0222834. doi: 10.1371/journal.pone.0222834 (PMC6762144; doi:10.1371/journal.pone.0222834)
Supplement: S1 File — Statistical Data. (PDF) [file pone.0222834.s001.pdf]

Supporting Information

Statistical Data

Supporting information for Table 10:

Table 10. Statistical results of the FSLs obtained with the Byrne equation (FLAC3D) for FSL=1 according to the Seed and Idriss model (1971) adapted by Boulanger and Idriss (2014).

Statistical Values of the FSL Result			
Average	Standard Deviation	Coefficient of Variation (%)	% > 1.3
1.26	0.36	28.78	≈50

Results FSL FLAC

Hyphotesis	Hyphotesis	HYPOTHESIS 1								
Earthquake	Earthquake	CI	SF	IV	TI	KC	PQ	M	CO	CP
M_w	M_w	6,60	6,61	6,53	7,35	7,36	7,50	8,80	8,30	8,30
Pga (m/s^2)	Pga (m/s^2)	1,22	2,00	5,90	0,85	1,60	3,50	1,40	3,40	6,70

FLAC (m)	Depth (m)	FSL	FSL	FSL	FSL	FSL	FSL	FSL	FSL	FSL
0,50	19,50	1,12	1,41	0,90	1,03	1,20	1,06	1,80	1,20	0,68
1,50	18,50	1,27	1,53	0,97	1,09	1,34	1,18	1,88	1,35	0,78
2,50	17,50	1,34	1,55	0,94	1,03	1,41	1,24	1,86	1,40	0,83
3,50	16,50	1,34	1,50	0,88	0,95	1,37	1,21	1,79	1,37	0,82
4,50	15,50	1,37	1,48	0,84	0,94	1,32	1,19	1,77	1,34	0,83
5,50	14,50	1,40	1,59	0,82	0,95	1,31	1,15	1,77	1,32	0,85
6,50	13,50	1,47	1,71	0,80	0,94	1,32	1,13	1,77	1,29	0,87
7,50	12,50	1,55	1,62	0,78	0,94	1,33	1,12	1,78	1,26	0,88
8,50	11,50	1,61	1,55	0,76	0,93	1,35	1,12	1,80	1,25	0,90
9,50	10,50	1,66	1,56	0,74	0,89	1,36	1,11	1,82	1,25	0,92
10,50	9,50	1,71	1,56	0,72	0,85	1,36	1,11	1,84	1,24	0,95
11,50	8,50	1,78	1,55	0,74	0,82	1,36	1,11	1,86	1,24	0,98
12,50	7,50	1,85	1,56	0,76	0,79	1,36	1,11	1,87	1,24	1,01
13,50	6,50	1,90	1,58	0,70	0,76	1,35	1,12	1,89	1,24	1,04
14,50	5,50	1,85	1,52	0,65	0,74	1,36	1,13	1,90	1,25	1,07
15,50	4,50	1,79	1,46	0,64	0,73	1,35	1,14	1,91	1,26	1,11
16,50	3,50	1,76	1,43	0,63	0,71	1,35	1,16	1,92	1,25	1,15
17,50	2,50	1,70	1,40	0,61	0,68	1,35	1,18	1,92	1,24	1,21
18,50	1,50	1,65	1,40	0,59	0,64	1,36	1,20	1,93	1,25	1,29
19,50	0,50	1,59	1,43	0,59	0,64	1,39	1,24	1,97	1,27	1,36
	**Table 10**	1,58	1,52	0,75	0,85	1,35	1,15	1,85	1,27	0,98
		**Average**	**1,26**	Variance	0,13	**Standard Deviation**	**0,36**	**Coefficient of Variation (%)**	**28,78**	

**Frecuency FSL**	**N.º Results**			
0,6	2,0	2,0	1%	1%
0,8	23,0	21,0	12%	13%
1	49,0	26,0	14%	27%
1,2	74,0	25,0	14%	41%
1,4	123,0	49,0	27%	68%
1,6	147,0	24,0	13%	82%
1,8	162,0	15,0	8%	90%
2	180,0	18,0	10%	100%
2,2	180,0	0,0	0%	100%
**Analyzed Data:**	**180**	180,0	100%	
		**FS**	**N.º**	**%**
		**>1,3**	**106**	**58**

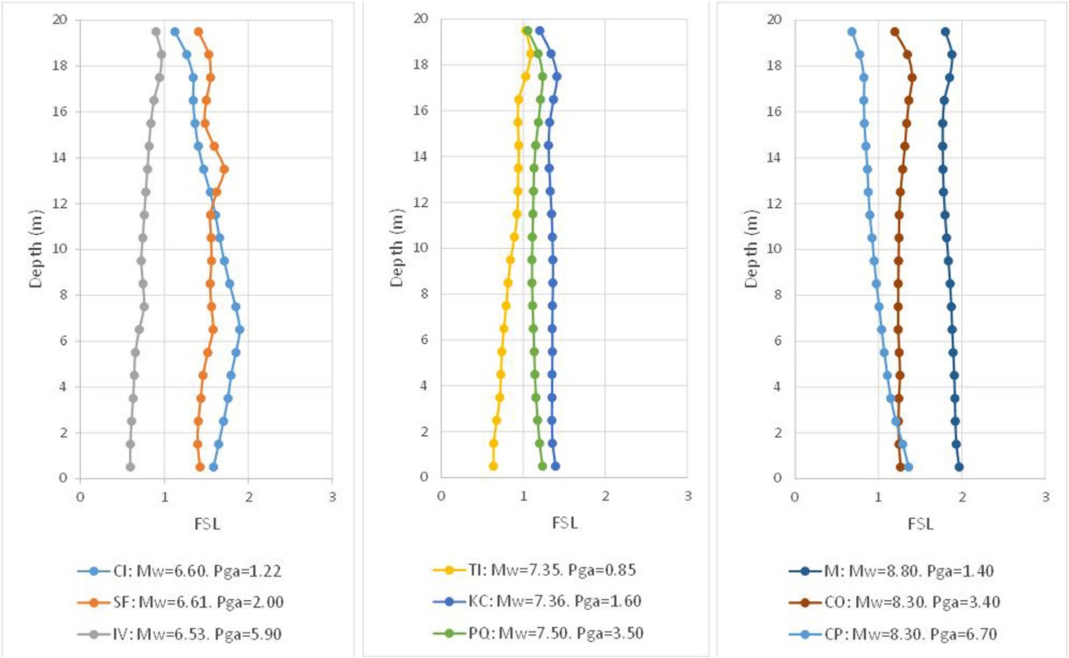

Fig 14. Distribution of the numerical factor of safety after the FSL=1.3 correction.

Supporting information for Table 12:

Table 12. Statistical results when the FSL is obtained with the corrected Byrne equation (FLAC3D) for FSL=1 according to the Seed and Idriss model (1971) adapted by Boulanger and Idriss (2014).

Statistical Values of the FSL Result			
Average	Standard Deviation	Coefficient of Variation (%)	% > 1.3
1.02	0.14	13.58	≈5

Results FSL FLAC

Hypothesis	Hypothesis	HYPOTHESIS 3								
Earthquake	Earthquake	CI	SF	IV	TI	KC	PQ	M	CO	CP
M_w	M_w	6,60	6,61	6,53	7,35	7,36	7,50	8,80	8,30	8,30
Pga (m/s^2)	Pga (m/s^2)	1,22	2,00	5,90	0,85	1,60	3,50	1,40	3,40	6,70

FLAC (m)	Depth (m)	FSL	FSL	FSL	FSL	FSL	FSL	FSL	FSL	FSL
0,50	19,50	0,81	0,89	1,10	0,94	1,08	1,01	1,02	1,37	0,71
1,50	18,50	0,85	0,90	1,13	0,97	1,24	1,13	1,09	1,30	0,80
2,50	17,50	0,87	0,90	1,12	1,00	1,26	1,16	1,08	1,24	0,85
3,50	16,50	0,87	0,91	1,12	1,05	1,17	1,11	1,03	1,19	0,85
4,50	15,50	0,90	0,89	1,06	1,09	1,14	1,08	1,02	1,15	0,85
5,50	14,50	0,95	0,83	0,96	1,04	1,11	1,06	1,02	1,11	0,85
6,50	13,50	1,00	0,81	0,90	0,97	1,08	1,03	1,04	1,10	0,87
7,50	12,50	1,03	0,94	0,88	0,98	1,08	1,02	1,06	1,09	0,89
8,50	11,50	1,09	1,10	0,86	0,99	1,08	1,01	1,06	1,09	0,92
9,50	10,50	1,15	1,01	0,84	0,92	1,08	1,02	1,07	1,10	0,95
10,50	9,50	1,17	1,03	0,82	0,87	1,08	1,01	1,08	1,11	0,99
11,50	8,50	1,18	1,14	0,79	0,85	1,09	1,01	1,10	1,11	1,02
12,50	7,50	1,18	1,04	0,77	0,85	1,09	1,02	1,11	1,11	1,06
13,50	6,50	1,17	0,94	0,75	0,86	1,09	1,04	1,12	1,09	1,11
14,50	5,50	1,14	0,91	0,72	0,86	1,09	1,07	1,12	1,08	1,14
15,50	4,50	1,12	0,90	0,70	0,87	1,09	1,05	1,13	1,09	1,17
16,50	3,50	1,10	0,93	0,67	0,89	1,08	1,04	1,13	1,10	1,21
17,50	2,50	1,09	0,94	0,65	0,89	1,08	1,05	1,16	1,10	1,26
18,50	1,50	1,05	0,93	0,63	0,92	1,09	1,07	1,18	1,12	1,33
19,50	0,50	1,05	0,93	0,63	1,02	1,11	1,10	1,18	1,16	1,44
	Table 12	1,04	0,94	0,86	0,94	1,11	1,05	1,09	1,14	1,01
		**Average**	**1,02**	Variance	0,02	**Standard Deviation**	**0,14**	**Coefficient of Variation (%)**	**13,58**	

Frecuency FSL	N.º Results			
0,6	0,0	0,0	0%	0%
0,8	10,0	10,0	6%	6%
1	62,0	52,0	29%	34%
1,2	171,0	109,0	61%	95%
1,4	179,0	8,0	4%	99%
1,6	180,0	1,0	1%	100%
1,8	180,0	0,0	0%	100%
2	180,0	0,0	0%	100%
2,2	180,0	0,0	0%	100%
**Analyzed Data:**	**180**	180,0	100%	
		**FS**	**N.º**	**%**
		**>1,3**	**9**	**5**

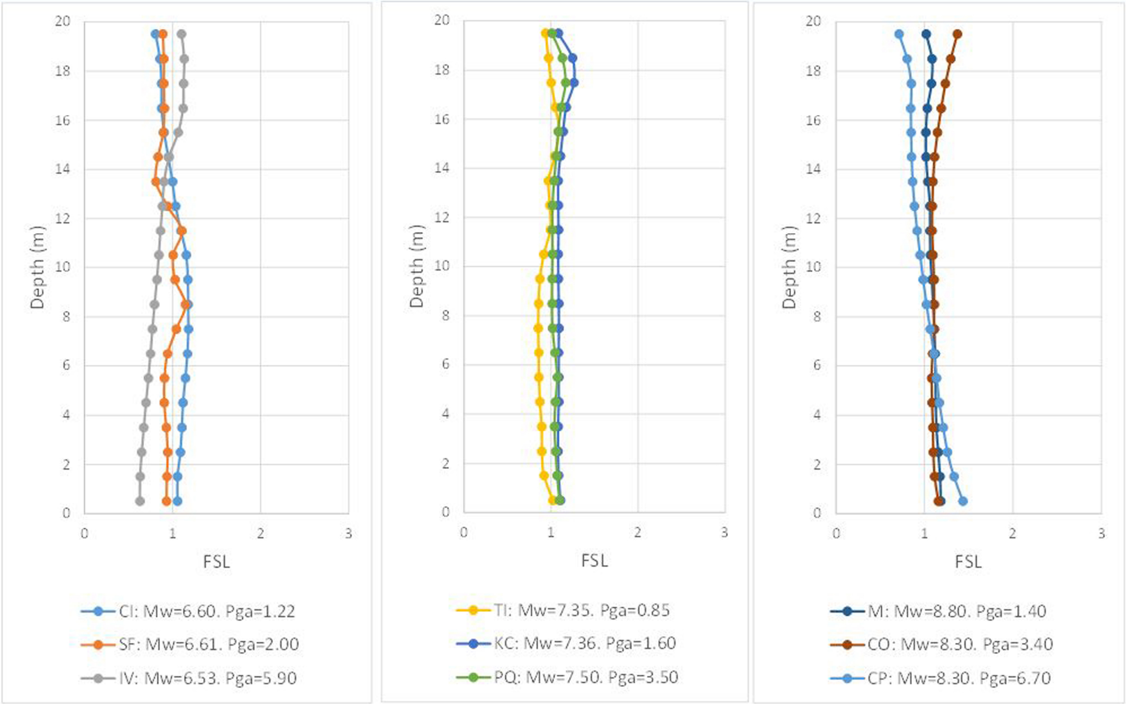

Fig 11. Numerical factor of safety vs. depth using $\boldsymbol{(N_1)_{60cs\_corr}}$ **verifying FSL=1, according to the Seed and Idriss model (1971) adapted by Boulanger and Idriss (2014).**

Supporting information for Table 13:

Table 13. Statistical results upon obtaining the FSL with the corrected Byrne equation (FLAC3D) for FSL=1.3 according to the Seed and Idriss model (1971) adapted by Boulanger and Idriss (2014).

Statistical Values of the FSL Result			
Average	Standard Deviation	Coefficient of Variation (%)	% < 1
1.29	0.26	20.41	≈10

Results FSL FLAC

Hypothesis	Hypothesis	HYPOTHESIS 4								
Earthquake	Earthquake	CI	SF	IV	TI	KC	PQ	M	CO	CP
M_w	M_w	6,60	6,61	6,53	7,35	7,36	7,50	8,80	8,30	8,30
Pga (m/s^2)	Pga (m/s^2)	1,22	2,00	5,90	0,85	1,60	3,50	1,40	3,40	6,70

FLAC (m)	Depth (m)	FSL	FSL	FSL	FSL	FSL	FSL	FSL	FSL	FSL
0,50	19,50	0,92	1,34	1,17	1,05	1,25	1,06	2,07	1,22	0,77
1,50	18,50	1,04	1,46	1,26	1,12	1,36	1,19	1,95	1,36	0,88
2,50	17,50	1,10	1,50	1,25	1,14	1,40	1,25	1,80	1,43	0,93
3,50	16,50	1,10	1,45	1,17	1,12	1,36	1,21	1,72	1,40	0,93
4,50	15,50	1,14	1,42	1,10	1,20	1,32	1,17	1,69	1,37	0,94
5,50	14,50	1,18	1,61	1,06	1,27	1,31	1,14	1,67	1,34	0,94
6,50	13,50	1,23	1,80	1,03	1,19	1,32	1,13	1,67	1,31	0,94
7,50	12,50	1,29	1,60	1,01	1,11	1,33	1,12	1,67	1,29	0,96
8,50	11,50	1,36	1,47	0,98	1,09	1,34	1,11	1,71	1,29	0,99
9,50	10,50	1,43	1,47	0,96	1,09	1,35	1,11	1,75	1,29	1,02
10,50	9,50	1,51	1,51	0,98	1,09	1,36	1,11	1,76	1,29	1,04
11,50	8,50	1,55	1,51	0,99	1,08	1,37	1,11	1,77	1,30	1,08
12,50	7,50	1,56	1,45	0,92	1,06	1,38	1,12	1,79	1,30	1,12
13,50	6,50	1,58	1,39	0,87	1,05	1,39	1,13	1,81	1,30	1,17
14,50	5,50	1,57	1,34	0,85	1,05	1,39	1,15	1,83	1,31	1,22
15,50	4,50	1,54	1,30	0,83	1,06	1,39	1,16	1,83	1,32	1,25
16,50	3,50	1,52	1,26	0,81	1,07	1,39	1,17	1,84	1,31	1,29
17,50	2,50	1,46	1,23	0,79	1,08	1,40	1,19	1,87	1,31	1,34
18,50	1,50	1,41	1,25	0,77	1,09	1,42	1,21	1,89	1,32	1,41
19,50	0,50	1,42	1,34	0,78	1,13	1,46	1,24	1,92	1,35	1,48
	**Table 13**	1,35	1,44	0,98	1,11	1,36	1,15	1,80	1,32	1,09
		**Average**	**1,29**	Variance	0,07	**Standard Deviation**	**0,26**	**Coefficient of Variation (%)**	**20,41**	

**Frecuency FSL**	**N.º Results**			
0,6	0,0	0,0	0%	0%
0,8	4,0	4,0	2%	2%
1	22,0	18,0	10%	12%
1,2	72,0	50,0	28%	40%
1,4	129,0	57,0	32%	72%
1,6	157,0	28,0	16%	87%
1,8	169,0	12,0	7%	94%
2	179,0	10,0	6%	99%
2,2	180,0	1,0	1%	100%
**Analyzed Data:**	180	180,0	100%	
		**FS**	**N.º**	**%**
		**>1,3**	**108**	**60**
		**<1**	**22**	**12**

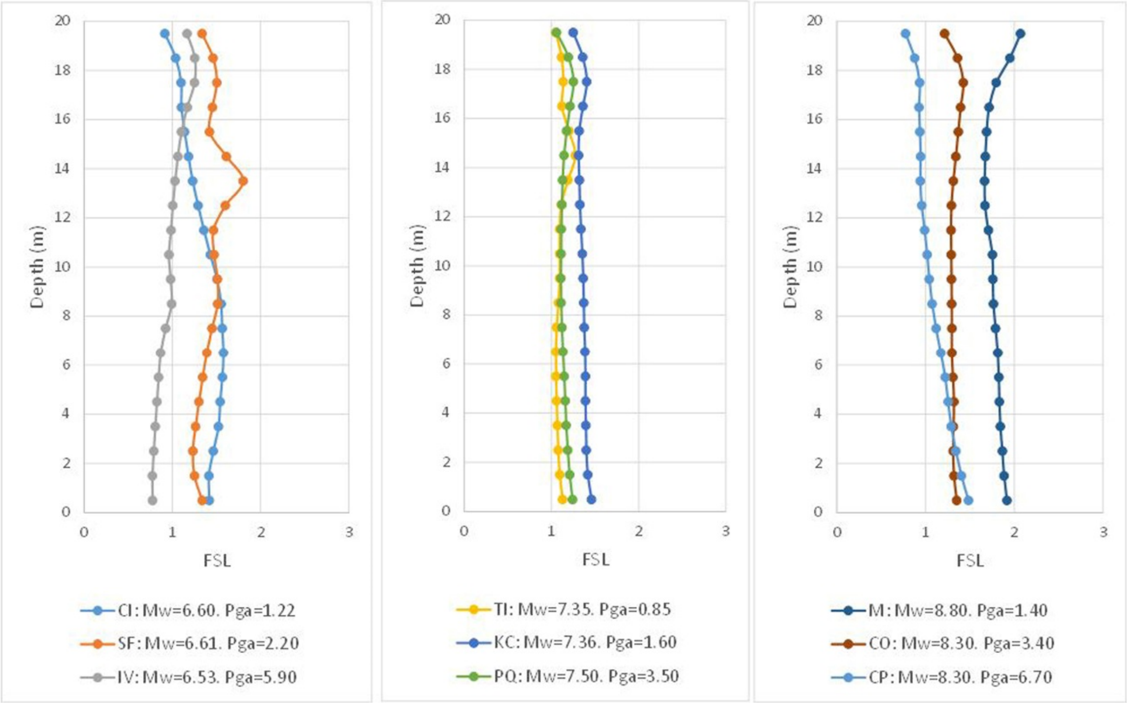

Fig 13. Numerical factor of safety vs. depth using the $(N_1)_{60cs\_corr}$ value verifying FSL=1.3 according to the Seed and Idriss model (1971) adapted by Boulanger and Idriss (2014).
